# Destabilizing polymorphism in cervid prion protein hydrophobic core determines prion conformation and conversion efficiency

**DOI:** 10.1371/journal.ppat.1006553

**Published:** 2017-08-11

**Authors:** Samia Hannaoui, Sara Amidian, Yo Ching Cheng, Camilo Duque Velásquez, Lyudmyla Dorosh, Sampson Law, Glenn Telling, Maria Stepanova, Debbie McKenzie, Holger Wille, Sabine Gilch

**Affiliations:** 1 Department of Ecosystem and Public Health, Calgary Prion Research Unit, Faculty of Veterinary Medicine, University of Calgary, Calgary, Alberta, Canada; 2 Center for Prions and Protein Folding Diseases, University of Alberta, Edmonton, Alberta, Canada; 3 Department of Biochemistry, University of Alberta, Edmonton, Alberta, Canada; 4 Department of Electrical and Computer Engineering, University of Alberta, Edmonton, Alberta, Canada; 5 Prion Research Center, Colorado State University, Fort Collins, Colorado, United States of America; Istituto Superiore di Sanità, ITALY

## Abstract

Prion diseases are infectious neurodegenerative disorders of humans and animals caused by misfolded forms of the cellular prion protein PrP^C^. Prions cause disease by converting PrP^C^ into aggregation-prone PrP^Sc^. Chronic wasting disease (CWD) is the most contagious prion disease with substantial lateral transmission, affecting free-ranging and farmed cervids. Although the PrP primary structure is highly conserved among cervids, the disease phenotype can be modulated by species-specific polymorphisms in the prion protein gene. How the resulting amino-acid substitutions impact PrP^C^ and PrP^Sc^ structure and propagation is poorly understood. We investigated the effects of the cervid 116A>G substitution, located in the most conserved PrP domain, on PrP^C^ structure and conversion and on 116AG-prion conformation and infectivity. Molecular dynamics simulations revealed structural de-stabilization of 116G-PrP, which enhanced its *in vitro* conversion efficiency when used as recombinant PrP substrate in real-time quaking-induced conversion (RT-QuIC). We demonstrate that 116AG-prions are conformationally less stable, show lower activity as a seed in RT-QuIC and exhibit reduced infectivity *in vitro* and *in vivo*. Infectivity of 116AG-prions was significantly enhanced upon secondary passage in mice, yet conformational features were retained. These findings indicate that structurally de-stabilized PrP^C^ is readily convertible by cervid prions of different genetic background and results in a prion conformation adaptable to cervid wild-type PrP. Conformation is an important criterion when assessing transmission barrier, and conformational variants can target a different host range. Therefore, a thorough analysis of CWD isolates and re-assessment of species-barriers is important in order to fully exclude a zoonotic potential of CWD.

## Author summary

Chronic wasting disease (CWD) is a prion disease which affects wild and captive cervids. Prion diseases are infectious neurodegenerative disorders, and the causative agent consists of abnormally folded prion protein termed PrP^Sc^. Prions replicate without genetic information, and their three-dimensional structure is thought to encode heritable information necessary to propagate using the cellular prion protein PrP^C^ as a substrate for conversion. In this study, we use *in vitro* and *in vivo* techniques to analyze the effect of a polymorphism at codon 116 (A>G) of the white-tailed deer prion protein on CWD prion conformation, propagation and pathogenesis. We observed differences in conformation, infectivity and seeding activity *in vitro* between CWD prions isolated from white-tailed deer encoding wild-type (116AA) PrP^C^ or 116AG-PrP^C^. In mouse bioassays conformational differences are retained, however, 116AG CWD prions resulted in significantly shortened incubation times upon passages. Molecular dynamics simulations suggest that the structure of 116G-PrP^C^ is more flexible, which is supported by an improved convertibility in an *in vitro* conversion assay. Altogether these data indicate the importance of a variation in the most conserved PrP domain, and highlight the relationship between PrP^C^ structural flexibility, prion conformation and conversion, and pathogenesis of prion disease *in vivo*.

## Introduction

Prion diseases are fatal neurodegenerative disorders including Creutzfeldt-Jakob disease in humans, bovine spongiform encephalopathy in cattle, scrapie in sheep and goats and chronic wasting disease (CWD) in cervids [1, 2].

According to the “protein only” hypothesis, prions are mainly, if not solely, composed of PrP^Sc^ [3], a misfolded isoform of the host-encoded prion protein, PrP^C^. PrP^Sc^ results from the conversion of PrP^C^ into a conformation enriched in β-sheets [4–6] which can act as a seed to bind and convert other PrP^C^ molecules. These are incorporated into a growing polymer [7, 8] which breaks into smaller oligomers, resulting in higher numbers of infectious nuclei. PrP^Sc^ is prone to aggregation and is partially resistant to proteases [9, 10]. Despite the absence of a nucleic acid genome, various prion strains have been identified, displaying specific biological properties [11]. Prion strains can be differentiated by incubation time, clinical signs of the disease and biochemical properties such as conformational stability of PrP^Sc^ within one host species [12–14].

CWD affects elk (*Cervus canadensis*), mule deer (*Odocoileus hemionus*), white-tailed deer (WTD; *Odocoileus virginianus*) and moose (*Alces alces*) [15, 16]. It is considered the most contagious prion disease with horizontal transmission favored by cervid interactions and environmental persistence of infectivity [17, 18]. The disease is present in North America, South Korea and, recently, has been identified for the first time in Europe in free-ranging reindeer (*Rangifer tarandus tarandus*) in Norway [19].

The transmission of prions is governed by a species barrier between species, or a transmission barrier within the same species [20, 21]. These barriers are regulated by the PrP primary structures of donor and recipient and emphasize the role of PrP structure in disease susceptibility. Species-specific allelic variants or polymorphisms were identified in cervid *Prnp*. WTD PrP can differ at residues 95, 96 and 116 [22–24]. Mule deer PrP has a unique polymorphism at residue 225. The contiguous residue 226 encodes the singular difference between deer and elk PrP and regulates strain selection [25]. In elk, PrP possesses a polymorphism at residue 132 [26, 27]. Most polymorphisms have in common that the presence of a single non-wildtype allele is associated with reduced susceptibility to CWD.

Transfer of prion strains between or within ruminant species expressing different PrP^C^ primary structures can result in the acquisition of novel transmission properties [26, 27]. This raises concerns on the zoonotic potential of prion agents from species consumed by human populations.

The polymorphism at residue 116 (A/G) in WTD is of particular interest. The wild-type (wt) genotype encodes an alanine (A) which is highly conserved among species [28]. The variant encodes a glycine (G); however, the influence of this mutation on CWD susceptibility is unclear. Whereas one study reports on no differences in the incidence of CWD in heterozygous WTD [22], others indicate that the 116G allele is found at a lower frequency in prion-infected than uninfected WTD [29].

Residue 116 (113 in human PrP) is localized in the central hydrophobic core (HC) of PrP^C^, which is the most conserved domain of PrP and critically involved in prion conversion [28, 30–32]. Mutations in the HC of human PrP are associated with heritable prion disease [33]. Therefore, we aimed to achieve a better understanding of potential structural changes in PrP^C^ caused by this single amino acid substitution, how it affects conversion efficiency and subsequently, CWD prion conformation, infectivity and pathogenesis *in vitro* and *in vivo*.

In wild cervids the most likely situation is that CWD prions from a certain host are transmitted to animals that express wt PrP. Our data demonstrate that prions from an animal with a PrP polymorphism can efficiently adapt to the wt PrP sequence. This may be due to a lower conformational stability of the WTD-116AG prions, which is likely the result of the predicted structural flexibility of PrP-116G, thus making it more prone to be converted. This is supported by an improved convertibility when recombinant PrP-116G is used as a substrate for conversion in real-time quaking induced conversion (RT-QuIC) assay. Our findings do not support earlier reports indicating a decreased susceptibility of WTD encoding the 116G allele to CWD infection, but indicate that investigations on adaptability and possible transmission of CWD prions with low conformational stability to non-cervid species are required.

## Results

### CWD isolates of individual WTD with different biochemical properties

We analyzed the *Prnp* genotypes of CWD-positive samples from Saskatchewan WTD (S1 Fig) and identified one isolate heterozygous at residue 116, encoding A and G at this site.

**Fig 1:**
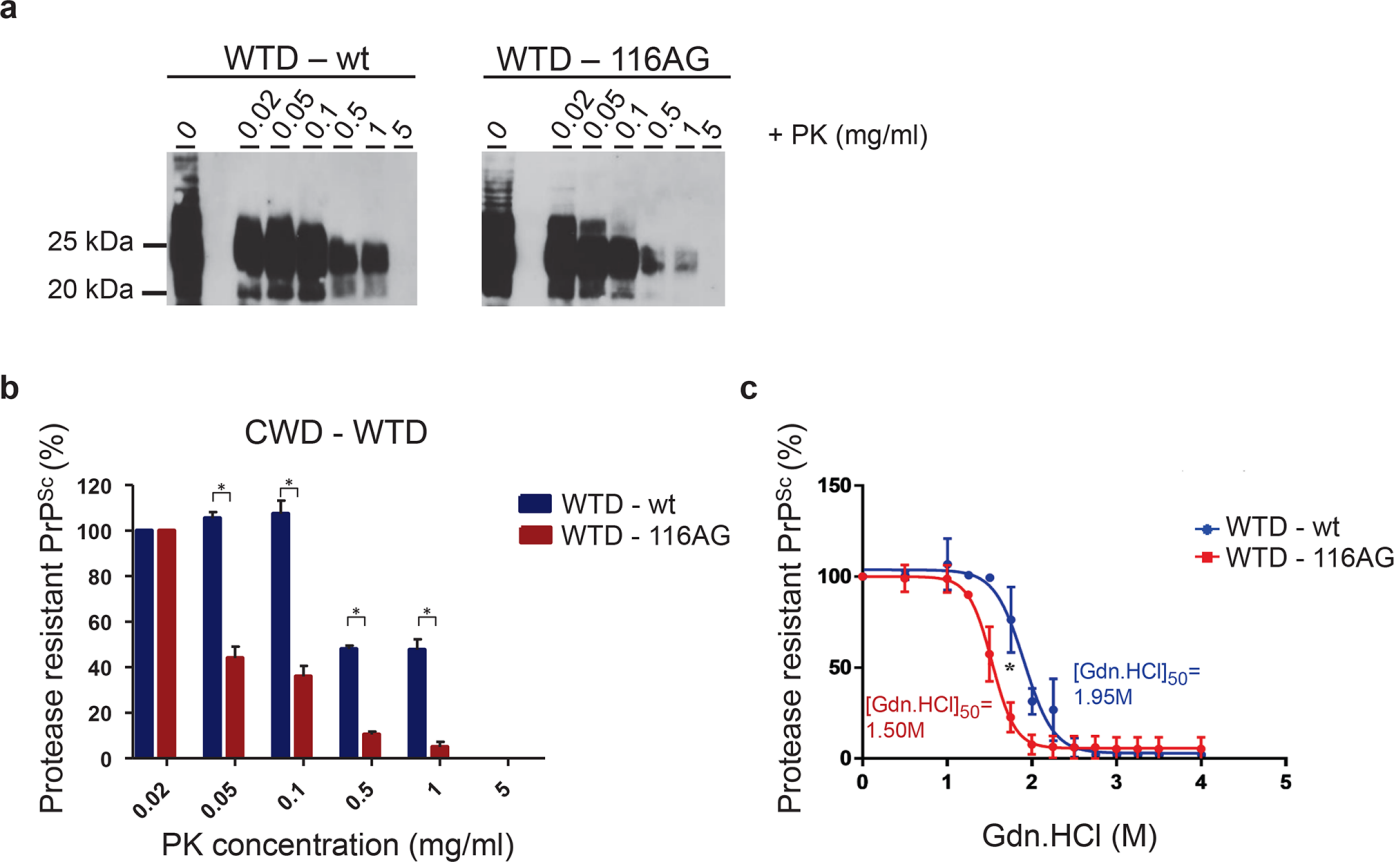
Biochemical properties of WTD isolates. Resistance to PK was assessed using different PK concentrations to digest wt (left panel) and 116AG (right panel) brain homogenates. (**a**) The PrP^res^ Western blot pattern was revealed using monoclonal antibody 4H11 and (**b**) assessed by densitometric analysis of PrP^res^ signals. *P <0.05 refers to differences between PK resistance of wt (blue bars) and 116AG (red bars) and evaluated using unpaired student’s t-test (GraphPad Prism software). (**c**) Conformational stability of wt (blue line) and 116AG (red line) assessed by densitometric analysis of PrP^res^ signals after guanidine denaturation followed by PK digestion. *P <0.05 refers to differences between [GdnHCl_1/2_] from best-fitted sigmoid curves evaluated using unpaired student’s t-test (GraphPad Prism software).

Single amino acid substitutions can substantially alter prion properties. Thus, we decided to assess the biochemical characteristics of wt and 116AG WTD CWD prions. We compared the proteinase K (PK) resistance of the two isolates by digestion of brain homogenates with different concentrations of PK (0 to 5 mg/ml). Western blot analysis indicated a dose-dependent decrease of the PrP^res^ signals (Fig 1A and 1B), significantly starting at 50 μg/ml of PK for the 116AG isolate and at a 10fold higher PK concentration for the wt isolate (Fig 1B).

Since differential PK resistance can arise from conformational variability, we compared the conformational stability of WTD isolates. The conformational stability assay (CSA) is a reliable tool to compare different prion isolates and distinguish prion strains [34]. It is used to determine the concentration of GdnHCl which is necessary to unfold 50% of the PrP^Sc^ (referred to as [GdnHCl_1/2_]). The [GdnHCl_1/2_] of 116AG and wt prions were significantly different (n = 5, *P < 0.05) with 1.95 M for wt and 1.5 M for 116AG (Fig 1C). A second WTD-wt isolate also showed a [GdnHCl_1/2_] of 2 M (S2 Fig). This experiment revealed a significantly higher conformational stability of wt PrP^Sc^ compared to 116AG PrP^Sc^.

### Reduced seeding activity, amplification efficiency and infectivity of 116AG prions *in vitro*

**Fig 2:**
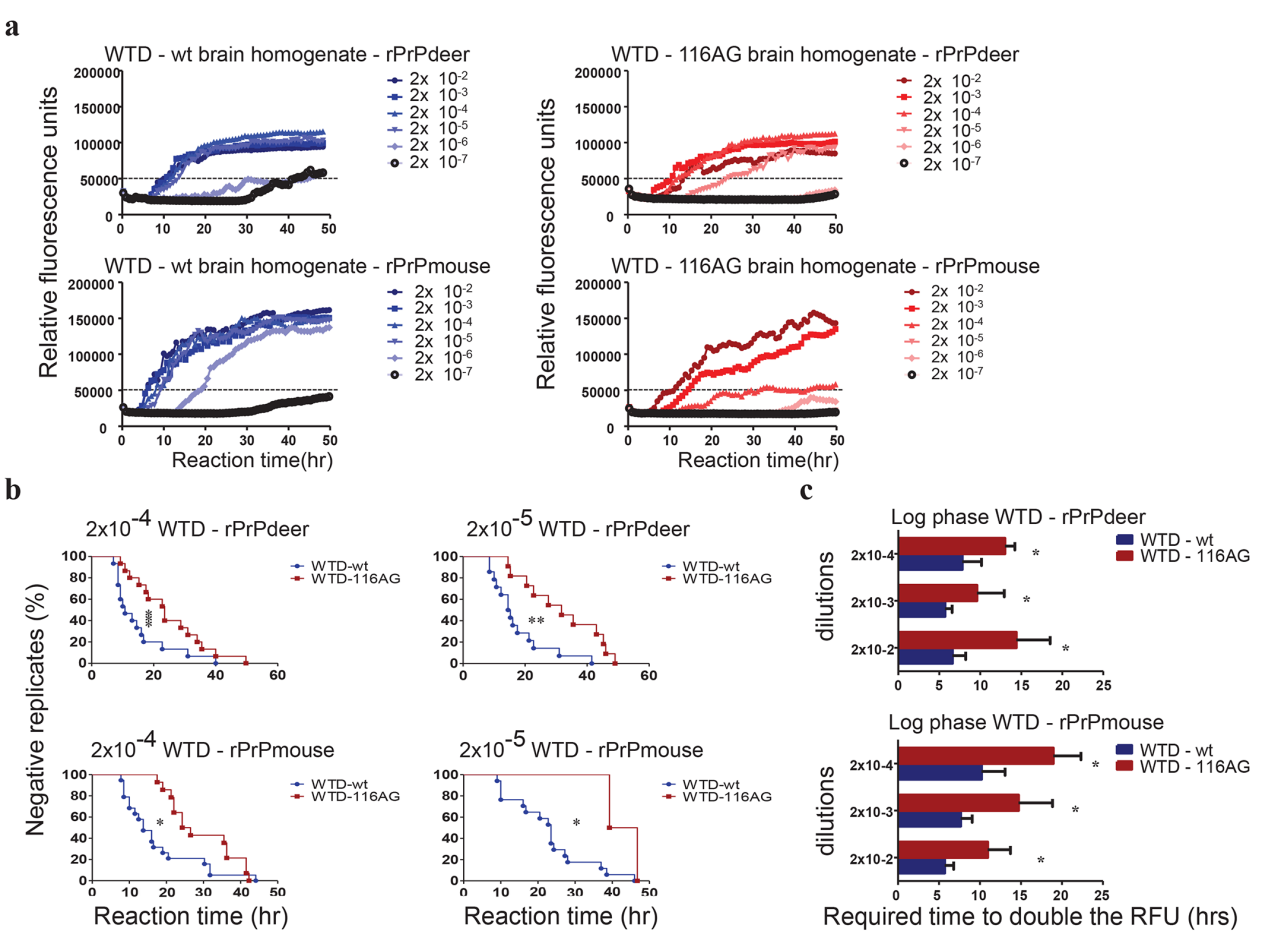
RT-QuIC analysis of WTD prion seeding and amplification characteristics using deer and mouse rPrP substrates. **(a)** The curves depict a representative RT-QuIC response of serially diluted (2x10^-2^ to 2x10^-7^) wt (left panels) and 116AG (right panels) brain homogenates using rPrP deer or mouse substrate. Fluorescence signals were measured every 15 min. The x-axis represents the reaction time (hours) and the y-axis represents the relative fluorescence units (RFUs), and each curve represents a different dilution. Mean values of four replicates were used for each dilution. The cut-off is indicated at app. 50,000 RFU based on the average fluorescence values of negative control +5SD. **(b, c)** The RT-QuIC responses of wt and 116AG were quantified by calculating different parameters: lag phase **(b)**, and log phase **(c)**. Mean values of 5 experiments with 4 replicates each were used and statistical analyses were evaluated using log-rank (Mantel-Cox) test for the lag phase (**b**) and unpaired t-test for the log phase (**c**). *P <0.05, **P <0.01 and ***P <0.005 refers to differences between WTD isolates (GraphPad Prism software).

We took advantage of RT-QuIC assay to compare the seeding and amplification characteristics of wt and 116AG prion seeds, respectively, using different recombinant PrP (rPrP) substrates as well as by looking into informative parameters such as endpoint dilution, lag and log phase [35]. Serial dilutions (2x10^-2^ to 2x10^-7^) of wt and 116AG brain homogenates were used to seed quadruplicate RT-QuIC reactions with deer or mouse rPrPs as a substrate (Fig 2A). Dilutions of which at least 50% of the replicates crossed a cut-off of approximately 50,000 RFU (relative fluorescence units) were considered positive.

Endpoint dilution analysis of wt and 116AG prions (5 independent experiments) showed that 116AG prions had a 100fold lower dilution endpoint than wt prions with both deer and mouse rPrP substrates (Fig 2A). To confirm that the lower dilution endpoints were related to the seeding activity of those isolates and not due to a different initial amount of PrP^Sc^ in each isolate, we serially diluted the two isolates after PK digestion. The subsequent Western blot analysis (S3 Fig) indicated that wt and 116AG brain homogenates harboured comparable amounts of PrP^res^.

Kinetics of conversion using 116AG prions as a seed resulted in extended lag and log phases compared to wt prions regardless of the rPrP substrate (Fig 2B and 2C). The graphs in Fig 2B illustrate the time individual replicates needed to reach the RFU threshold, which signifies the lag phase. The y-axis (Fig 2B) represents the percentage of replicates (individual RT-QuIC reactions) that did not yet reach the threshold. At the beginning of the reaction, 100% of the replicates were negative and over time, the percentage of those replicates decreased incrementally. The curves indicated that the lag phase in 116AG seeded reactions is significantly extended starting with the 2x10^-4^ dilution, regardless of rPrP substrate (Fig 2B).

Finally, we calculated the time needed for each condition (seed, substrate, dilutions) to double the RFU during the amplification phase of the reaction to characterize the log or elongation phase which signifies the efficiency of amyloid fibril formation. Independent of the rPrP substrate used, 116AG prion seeds resulted in a significantly longer log phase (1.5 to 2.5 times) compared to wt prion seeds (Fig 2C).

**Fig 3:**
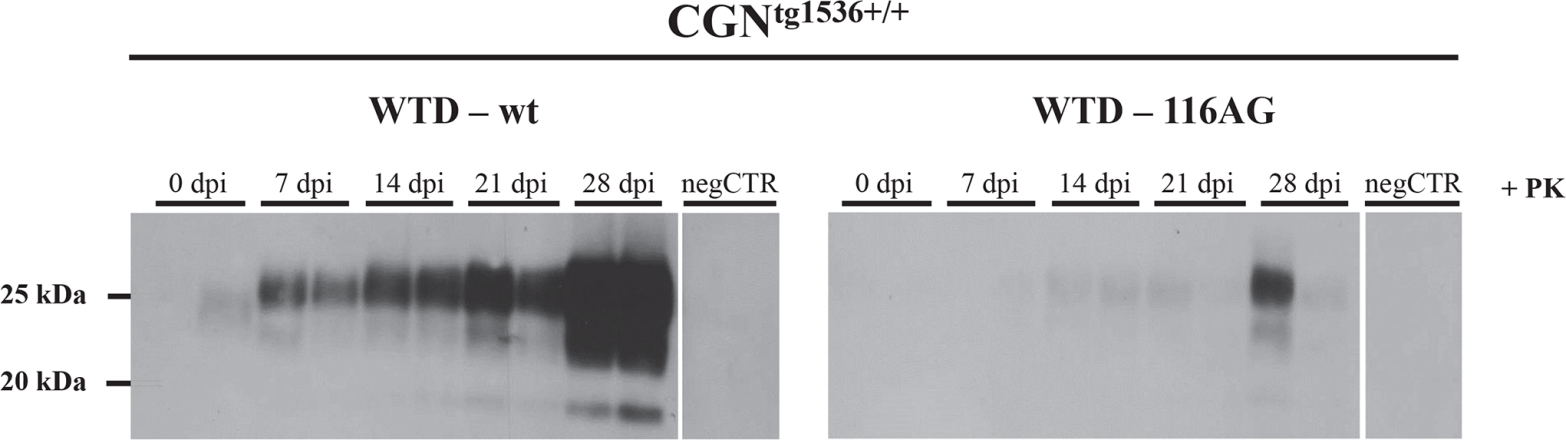
PrP^res^ detection in CGN^tg1536+/+^ primary neurons inoculated with WTD isolates. Kinetics of PrP^res^ accumulation after exposure of CGN^tg1536+/+^ to brain homogenates from terminally ill wt or 116AG WTD at a final concentration of 0.01% (wt/vol) were analysed by Western blot. Cells were lysed at different time points post infection (dpi) and 50 ug of protein were digested with PK. PrP^res^ was detected with monoclonal antibody 4H11. PrP^res^ accumulation was observed from 7 dpi to 28 dpi for the wt isolate (left panel), and from 14 dpi to 28 dpi for the 116AG isolate (right panel).

Besides, to investigate the infectivity of WTD isolates, we established primary cerebellar granular neuron cultures (CGN) derived from newborn tg1536^+/+^ mice [36]. We exposed CGN cultures to infected brain homogenates of either wt or 116AG WTD. Non-infected brain homogenates from WTD served as a negative control. We observed a progressive accumulation of PrP^res^ in infected CGN^tg1536+/+^ cultures (Fig 3). In CGN^tg1536+/+^ cultures infected with wt CWD prions, PrP^res^ accumulated as early as 7 days post infection (dpi) and progressively increased until the endpoint of the experiment (28 dpi; Fig 3, left panel) indicating a more efficient propagation compared to CGN^tg1536+/+^ cultures infected with 116AG prions (Fig 3, right panel). This result indicated that 116AG prions have a reduced infectivity in CGN cultures. Of note, this is the first evidence of primary neuronal cultures being infected with CWD agents.

### Increased incubation time and different histopathological features in tg1536^+/+^ mice inoculated with 116AG prions

We performed transmission studies in transgenic tg1536^+/+^ mice overexpressing deer PrP approximately six to eightfold [36]. For each group, at least nine female mice were inoculated intracerebrally with WTD brain homogenates. Tg1536^+/+^ mice inoculated with wt prions had a significantly shorter incubation period than mice inoculated with 116AG prions (*P* < 0.001, 241 ± 20 vs 301 ± 26; S4 Fig, Table 1.). Clinical presentation was similar in all mice; however, disease progression and duration of the clinical phase was clearly different, with one week in mice inoculated with WTD-wt and three months upon WTD-116AG infection.

**Table 1:** Intracerebral inoculation of transgenic mice that express cervid PrP with WTD isolates.

Isolate	First passage		Second passage	
	Tg1536^+/+^		Tg1536^+/+^	
	Mice with clinical disease	Incubation dpi ± SD	Mice with clinical disease	Incubation dpi ± SD
WTD–WT	**9/9**	**241 ± 20**	**5/5**	**248 ± 26**
WTD– 116AG	**13/13**	**301 ± 26**	**5/5**	**213 ± 38**

In addition, to assess differences between groups of mice inoculated with either of the two WTD isolates, coronal brain sections were examined histologically for spongiform changes and PrP^Sc^ aggregate distribution. Spongiosis and abnormal PrP aggregates were observed in cortex, hippocampus, corpus callosum, the habenular, thalamic and hypothalamic nuclei of two tg1536^+/+^ mice inoculated either with wt or 116AG prions. Despite a more intense staining upon inoculation of wt prions, PrP^Sc^ aggregates showed no consistent differences in terms of types of deposits between wt and 116AG inocula (S5A and S5B Fig). Vacuolation in the cortex (S5E and S5F Fig) of a mouse inoculated with 116AG prions was less pronounced compared to the vacuolation of a mouse inoculated with wt prions (S5C and S5D Fig).

### Biochemical differences of WTD isolates are retained upon first passage in tg1536^+/+^ mice

Similar to the characterization of WTD isolates, we analyzed the brain homogenates of the first passage in mice referred to as mWTD.

**Fig 4:**
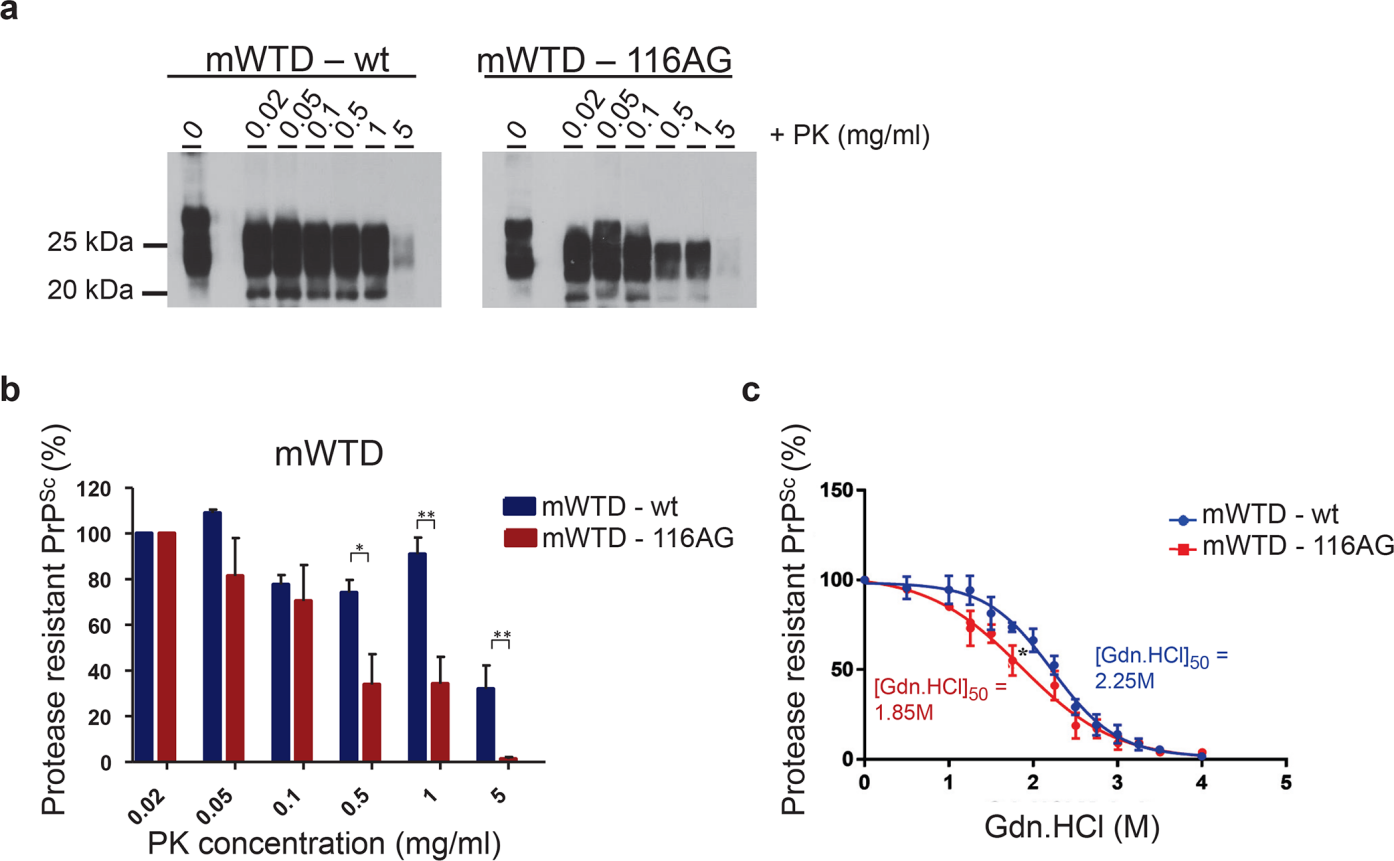
Biochemical properties of mWTD prions. Resistance to PK was assessed using different PK concentrations to digest mWTD-wt (left panel) and -116AG (right panel) brain homogenate. (**a**) The PrP^res^ Western blot pattern was revealed using monoclonal antibody 4H11 and (**b**) assessed by densitometric analysis of PrP^res^ signals. *P <0.05 and ** *P* <0.01 refers to differences between PK resistance of mWTD-wt (blue bars) and -116AG (red bars) and evaluated using unpaired student’s t-test (GraphPad Prism software). (**c**) Conformational stability of mWTD-wt (blue line) and -116AG (red line) was assessed by densitometric analysis of PrP^res^ signal after guanidine denaturation. *P <0.05 refers to differences between [GdnHCl_1/2_] from best-fitted sigmoid curves evaluated using unpaired student’s t-test (GraphPad Prism software).

Interestingly, assessment of PK resistance demonstrated that mouse brain extracts were slightly more resistant to PK compared to the WTD isolates regardless of the genotype of the inoculum (wt or 116AG; Fig 4A). However, mWTD-wt (blue bar) brain homogenates harbored PrP^Sc^ which resisted digestion with higher PK concentrations of 5 mg/ml compared to mWTD-116AG (red bar) prions (Fig 4B).

CSA analysis of mWTD prions was in line with the findings for the WTD isolates. Although the [GdnHCl_1/2_] of PrP^Sc^ of mWTD prions was increased compared to the original isolates, mWTD-wt and -116AG prions still differed in conformational stability, comparable to what was observed in the WTD isolates (Fig 4C). Again, mWTD-wt prions were significantly more stable than mWTD-116AG ([GdnHCl_1/2_] 2.25 M for mWTD-wt *vs*. 1.85 M for mWTD-116AG; P < 0.05).

**Fig 5:**
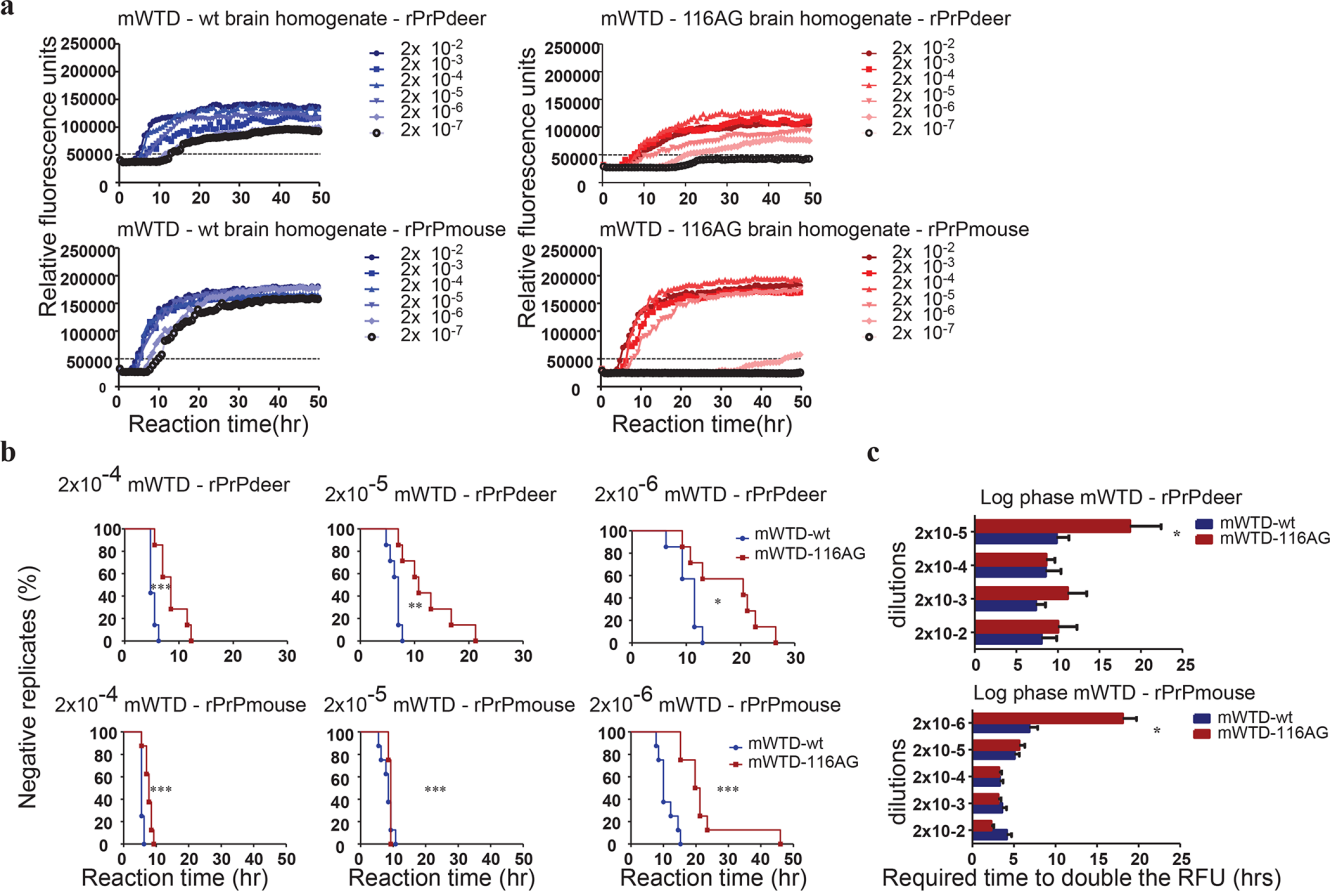
RT-QuIC analysis of mWTD brain homogenates using deer and mouse substrates. **(a)** The curves depict a representative RT-QuIC response of serially diluted (2x10^-2^ to 2x10^-7^) mWTD-wt (left panels) and -116AG (right panels) using rPrP deer or mouse substrate. Fluorescence signal was measured every 15 min. The x-axis represents the reaction time (hours), the y-axis represents the relative fluorescence units. Mean values of four replicates were used for each dilution. The cut-off is indicated at app. 50,000 RFU and is based on the average fluorescence values of negative controls +5SD. **(b, c)** The RT-QuIC responses of mWTD-wt and -116AG were quantified by calculating lag phase **(b)**, and log phase **(c)**. Mean values of 7 experiments with 4 replicates each were used and statistically evaluated using log-rank (Mantel-Cox) test for the lag phase (**b**) and unpaired t-test for the log phase (**c**). *P <0.05, **P <0.01 and ***P <0.005 refers to differences between mWTD isolates (GraphPad Prism software).

Next we performed RT-QuIC and endpoint dilution analyses, and it was evident that the seeding activities of mWTD-wt or mWTD-116AG prions (7 independent experiments) in mouse brain homogenates (Fig 5A) were higher than those of the original isolates (Fig 2A). In mWTD-wt seeded reactions, all dilutions up to 2x10^-7^ were positive regardless of the substrate. Brain homogenates containing mWTD-116AG prions had an at least 10fold lower dilution endpoint with both rPrP substrates (Fig 5A) despite comparable amounts of PrP^res^ (S6 Fig).

The conversion kinetics in reactions seeded with mWTD-116AG prions were different from reactions seeded with mWTD-wt prions. Similar to the original WTD isolate seeds, the lag phase when seeding the reactions with mWTD-116AG prions was extended from the 2x10^-4^ dilution, independent of the substrate (Fig 5B). However, except for one of the higher dilutions, the log phases of mWTD-116AG and mWTD-wt seeded reactions were similar with both substrates (Fig 5C). The fact that this difference in the log phase was apparent with the high dilutions only reflects a very efficient recruitment and conversion of rPrP molecules to the elongating amyloid fibrils once sufficient seeds are formed during the lag phase.

We infected CGN cultures derived from tg1536^+/+^ mice with mWTD-wt and mWTD-116AG or uninfected mice and assessed PrP^res^ accumulation over time (S7 Fig). Western blots showed a progressive accumulation of PrP^res^ in cultures infected with mWTD-wt starting at 14 dpi until the endpoint of the experiment (28dpi; S7 Fig, left panel). Results were consistent between duplicates up to 14 dpi, and despite a discrepancy between the duplicates at 21 and 28 dpi in CGN infected with mWTD-wt, this preliminary result indicates a more efficient propagation compared to cultures infected with mWTD-116AG prions, where PrP^res^ signals were weaker and increased slightly only after 21 dpi (S7 Fig, right panel).

### Significantly decreased incubation time of mWTD-116AG prions upon secondary passage in tg1536^+/+^ mice

Given the described differences in biochemical and amplification properties that were retained even on an identical host PrP genotype background, we wanted to verify whether this might reflect a novel CWD strain in the WTD-116AG isolate. Therefore, we performed a secondary passage of mWTD-wt or -116AG in tg1536^+/+^ mice (Table 1). Incubation times upon inoculation of tg1536^+/+^ mice with mWTD-116AG were significantly shorter than in the primary passage (*P* < 0.0001, 213 dpi ± 38 vs 301 dpi ± 26; Table 1 and S8 Fig). Surprisingly, 80% (4/5) of the mice inoculated with mWTD-116AG had a shorter incubation period compared to mice inoculated with mWTD-wt (S8 Fig). Disease progression of most of the mice inoculated with mWTD-116AG prions was shorter than observed upon first passage, except for one mouse with the longer incubation period. Notably, incubation times between primary and secondary passage of WTD-wt prions were comparable, with rapid disease progression (Table 1 and S8 Fig).

### Amyloid fibril formation of rPrP-116G is enhanced in RT-QuIC independent of the CWD-prion seed

**Fig 6:**
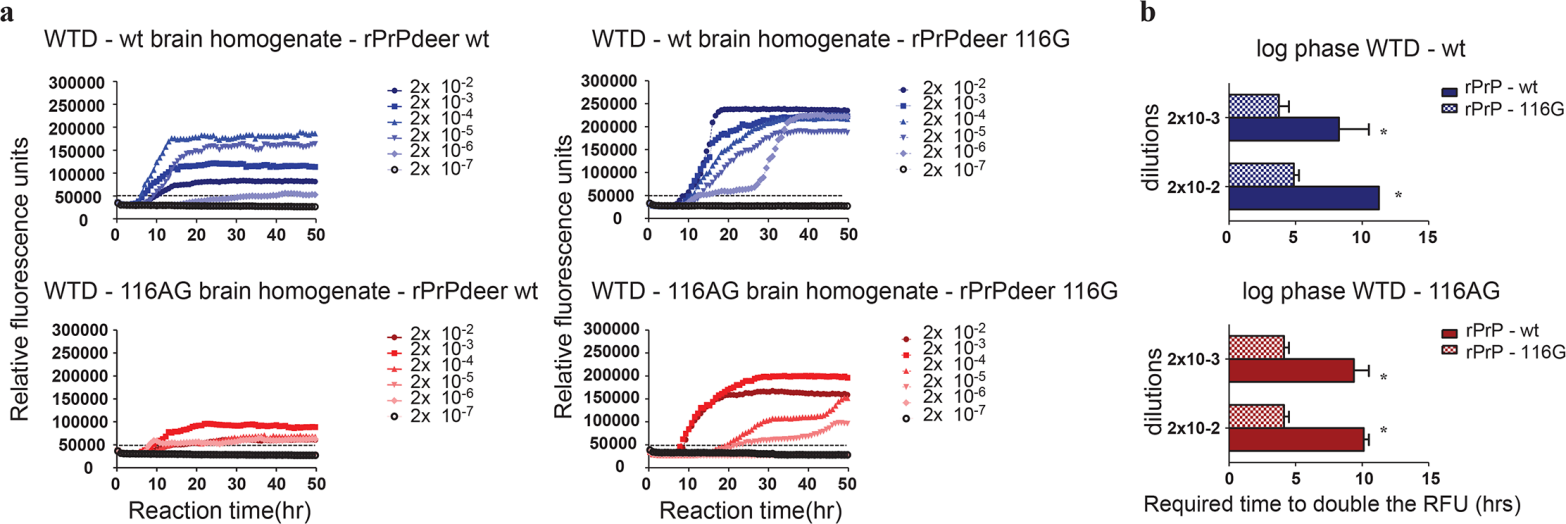
Efficiency of amyloid fibril formation using rPrP-116G substrate in RT-QuIC. **(a)** The curves depict a representative RT-QuIC response of serially diluted (2x10^-2^ to 2x10^-7^) wt (upper panels) and 116AG (lower panels) WTD brain homogenates using rPrP-wt (right panels) or rPrP-116G (left panels) as substrates. Fluorescence signals were measured every 15 min. The x-axis represents the reaction time (hours), the y-axis represents the relative fluorescence units. Mean values of four replicates were used for each dilution. The cut-off is indicated at app. 50,000 RFU and is based on the average fluorescence values of negative controls +5SD. **(b)** The RT-QuIC responses of wt- and 116AG-prions were quantified by calculating the log phase for each isolate amplified using rPrP-wt or rPrP-116G. Mean values of 3 experiments with 4 replicates each were used for each isolate and statistical analyses were done using unpaired t-test for the log phase (**b**).

To verify the conversion proficiency of the PrP-116G allele in seeded amyloid formation, we used the RT-QuIC technique to assess the ability of rPrP-116G to be converted by different CWD prion seeds. We used wt and 116AG prions (Fig 6A), respectively, as seeds and either rPrP-wt (116A) or rPrP-116G as substrates in RT-QuIC reactions. We found that rPrP-116G substrate was more efficiently converted (Fig 6A) than rPrP-wt. This is evident from an increased maximum RFU and a significantly shorter log phase for rPrP-116G substrate independent of the used CWD seed (Fig 6B). Despite the more efficient amyloid formation and conversion of rPrP-116G, which might indicate a greater ability for adaptation to any CWD seeds, we noticed that storage at -80°C negatively impacted RT-QuIC results with rPrP-116G substrate. This provided a hint that rPrP-116G is less stable than rPrP-wt and led us to perform molecular dynamics simulations of both deer PrPs for verification.

### 116G PrP shows more pronounced structural fluctuations than wt PrP

Computer simulations of protein dynamics provide valuable data to complement laboratory experiments [37]. The experimentally determined structure of the natively folded PrP was used to simulate conformational changes and physically realistic protein dynamics, including initial steps of misfolding [37]. The effect of pathogenic mutations on the molecular dynamics and conformation of PrP may be evaluated by applying mutations *in silico*. Thus, with tens of nanoseconds of MD simulation, the dominant contributions to intramolecular, atomic movements can be determined, making MD a useful and attractive method to analyze effects of single amino acid substitutions on PrP stability and identifying structurally flexible regions [38, 39].

**Fig 7:**
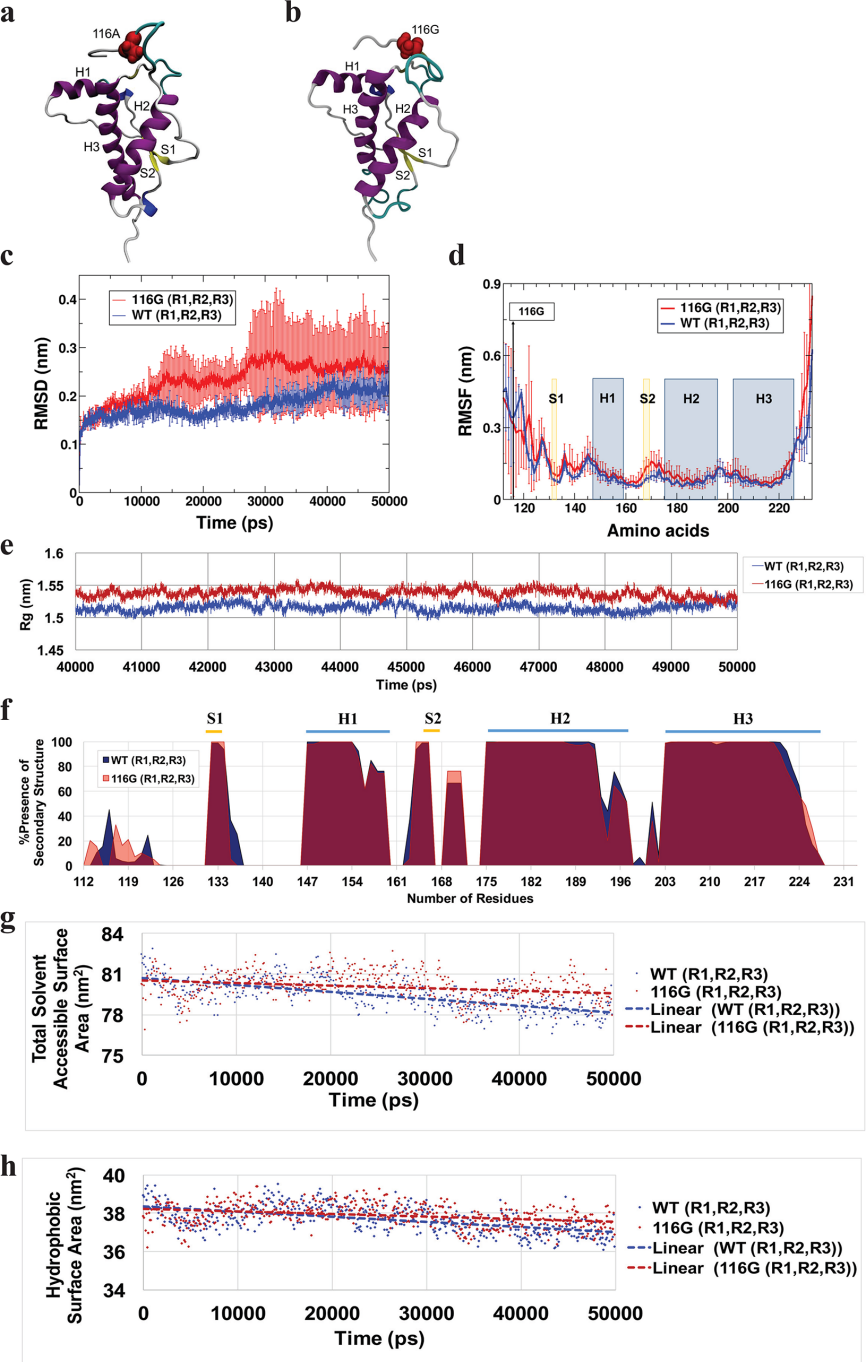
Molecular dynamics simulations. Comparison of wt and 116G PrP via MD simulations. (**a**) Cartoon representation of wild type PrP (residues 112 to 233): red spheres represent alanine in position 116. H1, H2 and H3 stand for helix one, two, and three, respectively. S1 and S2 stand for β-strands one and two. (**b**) Cartoon representation of 116G PrP: red spheres represent glycine in position 116. (**c**) RMSD plot for the folded domains of both wt (blue) and 116G polymorphism (red). (**d**) RMSF plot comparing wt (blue) and 116G polymorphism (red). The location of the polymorphism at residue 116 is shown with a black line and a label. The yellow and blue bars highlight the location of individual β-strands and α-helices, respectively. Both graphs show the average of three rounds (R1, R2, R3) of simulation. (**e)** Radius of gyration (Rg) values for both wt (blue) and the 116G polymorphism (red) as a function of simulation time. Both graphs are the average of three rounds (R1, R2, R3) of simulation. (**f**) Per-residue percentage of dominant secondary structure for the last 20 ns of production simulation. The blue and red graphs represent wt and the 116G polymorphism, respectively. The location of known secondary structure elements are shown using yellow and blue bars (top), on the crystal structure of deer prion protein (PDB ID: 4yxh [66]. (**g**) Solvent accessible surface area for both 116G PrP and wt PrP. (**h**) Hydrophobic surface area for both 116G and wt. (**g**) and (**h**) both show the average of three rounds (R1, R2, R3) of simulation and the dashed lines represent the linear fit for the solvent accessible surface area and the hydrophobic surface area values, respectively.

For both wt (Fig 7A) and the 116G polymorphism (Fig 7B) simulations were done with three different starting configurations of atom velocities (labeled R1, R2 and R3) to ensure reproducibility and consistency of the results. The final trajectories were analyzed specifically focussing on the structural stability and dynamic of both systems.

The root mean square deviation (RMSD) of the backbone Cα atoms for the folded domains of wt and the 116G polymorphism were calculated and plotted in Fig 7C. The graph for the RMSD represents the average of the three independent MD runs over time, with the error bars corresponding to the standard deviation between the separate simulations. Comparing the RMSD values, it became apparent that the 116G polymorphism had higher RMSD values than wt and a much larger variation between individual simulations, indicating that the 116G conformation has more pronounced structural fluctuations than those of wt PrP.

Another parameter determining the dynamic stability of a protein is the root mean square fluctuation (RMSF) value, which represents the flexibility of a specific residue around its average position. We calculated RMSF values for all six simulations and plotted averaged results for wt and 116G, with the error bars corresponding to the standard deviation between the separate simulations. Fig 7D shows that the 116G polymorphism affects the RMSF of most residues, but particularly those close to the 116G exchange, making the structure of 116G PrP less stable. In particular, we observed a substantially increased destabilization around the second β-strand and in loops LS2H2, LH1S2, LS1H1.

### wt PrP is more compact compared to 116G

By calculating the radius of gyration (Rg) we determined the effective size of the proteins throughout their simulation (50 ns). This approach gave us a picture of the protein’s folding behaviour. To compare the packing behaviour of both wt and 116G, the radius of gyration of all Cα atoms was calculated and plotted. Fig 7E presents the Rg values for the final 10 ns of the simulation. It is readily apparent that the 116G polymorphism had a generally larger Rg value compared to the wt, implying that the 116G substitution affects the conformation of the whole protein. It is well-known that protein folding is an important contributor to protein stability, thus the more compactly folded wt protein may be more stable than the 116G polymorphism.

### 116G polymorphism exhibits signs of structural disruption

The secondary structure (SS) plugin of the VMD analysis package [40] was used to assign the SS elements for all rounds of simulation. Subsequently, the dominant SS element for each residue for the last 20 ns of simulation was evaluated. However, only the percentage of α-helices, β-strands, and β-bridges was plotted (Fig 7F, graphs represent the average of SS assignments from all three simulations for each system, with a more detailed analysis for all three simulations shown in S9).

New SS elements appeared in the N-terminal regions of both wt and 116G polymorphism (residues 112 to 125). In wt the N-terminal SS consisted predominantly of α-helices, while in 116G the SS elements were reduced by about ~12% and consisted mostly of β-strands and β-bridges. Furthermore, the first β-strand (S_1_) was longer in wt PrP compared to the 116G polymorphism (residues 131 to 137 and 131 to 135, respectively). In contrast, the lengths of the first α-helix (H_1_) and the second β-strand (S_2_) were very similar in both systems. Another new SS element, which was seen in both wt and 116G involved residues 168 to 172. In wt PrP residues 169 to 171 were mostly α-helical (~70%) and to a lesser degree (~30%) in a β-strand / β-bridge conformation. However, in 116G these residues were seen in equal proportions in both conformations (~50% for each), meaning they were more likely to be in a β-strand conformation than for wt PrP. The second helix (H_2_) was very much preserved in both wt and 116G with a partial disruption of its final residues (residues 193 to 198). The same trend was seen in the third helix (H_3_), residues 223 to 228 had lost their helical structure for about 50% of the time in the simulation. Also, residues 200 to 202 had gained α-helical structure with an elongated H_3_ in both wt and 116G.

An overall assessment of the SS throughout the simulations showed that PrP with the 116G polymorphism exhibited signs of structural disruption compared to wt PrP. Meaning the amino acids in wt PrP were more likely to be folded (α-helix or β-sheet), rendering the protein more stable. In addition, the new SS elements (residues 112 to 125, and 168 to 172) of 116G preferred to adopt a β-structure (β-strand / β-bridge), while wt PrP preferred α-helical structure. This finding matched previous MD studies indicating that mutated forms of the prion protein can adopt higher β-sheet content [41].

### Increase in the accessible surface area of 116G polymorphism

The averages for the total solvent accessible surface area (SASA) for wt and 116G PrP were computed over 50 ns of simulation and plotted in Fig 7G. 116G PrP had a slightly larger SASA value compared to wt PrP, indicating that the polymorphism made the structure of PrP more solvent exposed. In addition, a minor increase in exposure of hydrophobic residues of the PrP structure was seen in 116G compared to wt (Fig 7H). An increase in hydrophobic exposure is an indicator of weaker interactions between solvent and protein, thereby rendering a protein more likely to undergo self-assembly [41].

## Discussion

Polymorphisms in the prion protein gene *Prnp* are important modulators of susceptibility to and pathogenesis of prion diseases [42]. Here, we characterized the effect of the 116A>G polymorphism identified in a CWD isolate from a 116AG heterozygous WTD. We discovered that 116AG prions had a lower conformational stability, seeding activity in RT-QuIC and infectivity in CGN and mouse bioassays than wt (116AA) prions. Biochemical differences and certain amplification characteristics were retained following passage in transgenic mice overexpressing wt deer PrP. This may indicate the isolation of diverse CWD strains arising from wt and 116AG CWD isolates. Notably, this is the first study revealing differences in conformational stability between PrP^Sc^ of CWD isolates, and moreover, even after passage in mouse models. Stability of PrP^Sc^ is associated with prion conformation and a widely accepted hallmark of strain variability [11, 43, 44]. Furthermore, we determined quantitative parameters for RT-QuIC to characterize seeding and amplification characteristics and established for the first time CGN cultures as a novel cell culture model for CWD infection.

Previous studies have been contradictory with respect to the relationship between conformational stability of PrP^Sc^ and incubation periods [45–47]. Legname *et al*. showed an inverse correlation between stability of PrP^Sc^ aggregates and incubation time [47]. Efficient cell-free conversion of ovine PrP^C^ into protease resistant forms was correlated to polymorphisms conferring susceptibility to scrapie [48].

We demonstrate that upon second passage of mWTD-116AG prions, lower conformational stability was correlated to a shorter incubation time. The fact that the differences in PK resistance and conformational stability between wt and 116AG prions were retained upon passaging indicates that the delay in incubation period observed in the primary transmission of 116AG probably was due to the single amino acid difference between inoculum and host PrP, creating a transmission barrier. This pattern was reflected by the duration of the log phase in RT-QuIC indicative of elongation of amyloid fibers, which was attenuated and almost equal between mWTD-wt and -116AG seeded reactions, in contrast to the significantly prolonged log phase of the WTD-116AG seeds in the WTD isolate. Heterozygosity of WTD could also lead to a co-existence of two conformers of PrP, wt and 116G, thus leading to competition and/or selection during prion fiber formation. Propagation in the CGN model still differed between the two mWTD prion inocula. This apparent discrepancy to the shortened incubation time *in vivo* and log phase in RT-QuIC validates that onset of clinical disease not necessarily correlates with *in vitro* propagation and is influenced by regional or host factors, or differential toxicity of prions. It also might reflect the differences observed in the lag phase of the RT-QuIC reactions in that sufficient seed formation is delayed and the mWTD-116AG infected CGN cultures not yet reached the exponential propagation phase.

The 116 (A>G) polymorphism introduces a variation into the highly conserved hydrophobic core region (HC) of PrP [28]. One mutation in this domain (117A>V) causes a heritable prion disease of humans where disease progression was not associated to amyloid formation [33]. The HC can facilitate PrP^C^-PrP^Sc^ interaction and the formation of β-sheet structure [49, 50], can act as a hinge region involved in PrP^C^-PrP^Sc^ conversion [30, 31] and is of importance for the interaction with lipids which may facilitate the conversion of PrP^C^ into PrP^Sc^ [32].

Intriguingly, using rPrP-116G substrate in RT-QuIC resulted in a more efficient conversion independent of the used seed compared to the rPrP-wt substrate. Substitution of A with G replaces a hydrophobic, aliphatic residue (A) with a more polar residue (G). Thus, PrP^C^-wt may be capable of better van der Waals packing. In fact, MD simulations revealed that the structure of PrP^C^-116G is less stable than PrP^C^-wt and exhibits structural disruption and more solvent accessible surface area. This indicated a more flexible structure of PrP^C^-116G possibly more prone to conversion, resulting in prions with a lower conformational stability and PK resistance of original and mouse-passaged WTD-116AG prions. Interestingly, MD analysis indicated that the 116G allelic variant has a higher probability to form β-strands in the rigid loop region. The structural rigidity of this PrP domain has been discussed to be predictive of susceptibility to CWD infection, with higher flexibility of the loop conferring resistance [51]. Altogether, our data argue against a reduced susceptibility of WTD carrying the 116G allele, based on the increased effectiveness of amyloid formation of rPrP-116G, the predicted higher propensity of β-strand elements in the rigid loop region, and the new β-strand elements in N-terminal PrP^C^-116G (residues 112 to 125), predicted to be mainly α-helical in PrP^C^-wt. As a note of caution, with cell-free conversion assays dose, prion strain and route of infection cannot be considered, which all affect the ability of PrP^C^ and PrP^Sc^ to interact and the progression of clinical disease.

CWD strains are poorly documented. To date, there are few studies describing the existence of CWD strains [25, 52] and the emergence of CWD strains with novel transmission properties [53]. First evidence of CWD strains was provided when transgenic cervid mice inoculated with a variety of CWD isolates revealed the identification of two CWD strains, termed CWD1 and CWD2 [25]. These two strains, with different incubation periods, exhibited indistinguishable biochemical properties of PrP^Sc^. Interestingly, primary transmission of elk isolates displays either CWD1 or CWD2 profiles, but deer tend to harbor a CWD1/CWD2 mixture which was attributed to the singular difference between elk (226E) and deer (226Q) PrP and strain mutation [25].

Oral transmission of wt/wt deer prions into WTD expressing different PrP^C^ primary structures (wt/wt, S96/wt, H95/wt or H95/S96) [54] resulted in emergence of a strain (H95^+^) with novel transmission properties in deer expressing H95-PrP^C^ [53]. Transmission of these deer isolates into transgenic mice expressing deer wt (tg33) or S96 PrP^C^ (tg60) resulted in differential strain propagation and revealed that H95/wt and H95/S96 deer accumulated a strain mixture (Wisc-1 and H95+) while wt/wt and S96/wt carry the Wisc-1 strain mostly [53]. These results suggest evolution of cervid strains occuring by transmission between hosts expressing different PrP^C^ primary structures. In contrast to this, *Prnp*-independent alterations of CWD strains upon transmission to different host species expressing the same PrP^C^ were reported, highlighting the relevance of host-specific factors [55].

Our study favors the emergence of a new strain based on structural differences of PrP^C^-116G that could overcome the mutation effect of 226Q [25] and adapted efficiently to the wt PrP structure upon secondary passage in tg1536^+/+^ mice.

Given the frequencies of non-wt alleles in cervids, transmission of CWD prions from animals expressing PrP allelic variants to animals harboring wt PrP is the most likely scenario among free ranging cervids. We suggest that CWD transmission between cervids expressing distinct PrP^C^ molecules can introduce prion conformational variability that may modify transmission properties. Nevertheless, controlled oral inoculation as well as the generation of mouse models expressing PrP^C^-116G will be required to determine the degree to which the 116G allele affects strain properties and susceptibility to CWD infection.

Our data add new insights into the association between structural stability of PrP^C^, conversion efficiency *in vitro*, conformational stability of CWD prions and infectivity and adaptation *in vitro* and *in vivo*. It improves our understanding of the impact of single amino acid substitutions on predicted structural properties and convertibility, suggesting that higher flexibility aligns with improved conversion but results in prions with a less stable conformation. However, this instability may enhance adaptation to PrPs of non-cervid species. Although until to date CWD prions have failed to transmit disease to transgenic mice expressing human PrP [56, 57] as well as to cynomolgus macaques [58] suggesting that humans are resistant to CWD, this risk cannot be totally excluded. CWD prions arising from cervids expressing allelic variants that are converted to conformationally instable prions could be the missing link to determine the real zoonotic potential.

## Materials and methods

### CWD isolates

All isolates used in this study were prepared as 10% (W/V) brain homogenates in phosphate-buffered saline pH 7.4 (PBS) using a dounce homogenizer. Aliquots were stored at -80°C. The wt isolate was obtained by experimental oral infection [54], and the 116AG from a free ranging animal at the terminal stage of disease.

### Ethics statements

All work with animals was performed in compliance with the University of Calgary Animal Care Committee under protocol numbers AC14-0025 (inoculation) and AC14-0117 (primary cultures) and CCAC guidelines. The University of Calgary Animal Care Committee approved the study. The transgenic mouse line tg(CerPrP132M)1536^+/+^ overexpressing wt deer PrP [34, 36] was used to propagate the two WTD isolates. The relative level of PrP overexpression in the brain of these mice was about six to eightfold [36].

### Mouse bioassays

Six to eight weeks old female mice were anaesthetized and inoculated with 20 μl of a 1% brain homogenate of either of the two WTD isolates in the right parietal lobe using a 25 gauge disposable hypodermic needle. Mice were initially monitored weekly and daily when progressive clinical signs of prion disease were evident. At the experimental endpoint, animals were anaesthetized before being euthanized by CO_2_ overdose and their brains were collected and frozen at -80°C. For the second passage, animals were inoculated with 20 μl of a 1% brain homogenate of either of the two mWTD prions, corresponding to first passage mouse brain homogenate.

### Incubation time

Incubation time was expressed as the mean value of the days post inoculation (dpi) for all mice that tested positive for PrP^Sc^ after PK digestion (PrP^res^). The statistical analysis of transmission experiments was performed using GraphPad Prism (version 5) software and using the Mann-Whitney test.

### Histopathology

Brain tissues of one mouse each inoculated with either of the WTD isolates were fixed and paraffin embedded for histopathology. Coronal brain sections were performed and slices (4 to 6 μm thick) were stained with hematoxylin and eosin to evaluate the sections for spongioform degeneration and immunostained for PrP^Sc^ deposition using mAb BAR224. Briefly, brain slides were pretreated with high-pressure autoclaving (2.1 × 10^5^ Pa) for 30 min in citric acid (10 mM), pH 6.0, at 121°C, followed by treatment with 98% formic acid for 30 min and 4 M guanidine thiocyanate for 2 h at room temperature. Tissue sections were scanned with a NanoZoomer 2.0RS scanner (Hamamatsu Photonics) and analyzed using NanoZoomer digital pathology software (Hamamatsu Photonics).

### Anti-PrP antibodies

The anti-PrP monoclonal mouse antibodies used in this study were 4H11 (1/500; [59]) or BAR224 (1/10,000; Bertin Pharma, Fr).

### PrP^res^ Western blot detection

For PrP analysis in brain extracts, brain homogenates (3 different animals) prepared in PBS were either not digested or treated with different concentrations of PK (0 to 5 mg/ml; VWR, Ca) as indicated for one hour at 37°C. The reaction was terminated by adding 1X pefabloc proteinase inhibitor (VWR, Ca). Fifty μg of protein were separated by sodium dodecyl sulphate polyacrylamide gel electrophoresis (SDS-PAGE), and then electrophoretically transferred to PVDF membranes (Millipore, Ca). PVDF membranes were probed using anti-PrP monoclonal antibodies followed by horseradish peroxidase-conjugated goat anti-mouse IgG antibody (Sigma, Ca) and developed using ECL-plus detection (Amersham). Images were acquired on X-ray film (Super Rx; Fujifilm) or by using a digital imaging system (Alpha Innotech, FluoriChemQ). FluoChemQ software (Alpha Innotech) was used to quantify and determine the relative values of PrP^res^ signals.

### Conformational stability assay (CSA) of PrP^Sc^

CSA was performed as previously described [34] with slight modifications. Briefly, 10% brain homogenates from WTD or mice upon primary passage of the WTD isolates (3 different animals) were incubated with various concentrations (0 to 4 M) of GdnHCl (Sigma, Ca) for 1 hour at 20°C under shaking conditions (450 rpm). Then samples were treated with 50 μg/ml of PK for an additional hour at 37°C and the reaction was stopped by adding 1X pefabloc proteinase inhibitor. The samples were then subjected to Western blot and PrP^res^ signals were quantified as described above. The relative values of PrP^res^ (5 independent experiments) were plotted as a sigmoid curve against the GdnHCl concentration using GraphPad Prism (version 5). The GndHCL concentration required to denature 50% of PrP^Sc^ [GdnHCl_1/2_] was deduced from these curves. The statistical analysis to compare the different isolates was performed using GraphPad Prism (version 5) software using unpaired student’s t-test.

### Preparation of recombinant PrP (rPrP) substrate

In this study we used the mature forms of deer, wt (aa 24–234; construct kindly provided by B. Caughey, NIH Rocky Mountain Laboratories, Hamilton, MT), 116G (aa 24–234) or mouse (aa23-231) PrP cloned into pET expression vectors and expressed in *E*. *coli* Rosetta using the Express Autoinduction System (Novagen). The 116G mutant was created by site-directed mutagenesis of the wild-type deer PrP in the pET plasmid. Inclusion bodies were prepared using the Bug Buster reagent (Novagen) and solubilized in lysis buffer (guanidine-HCl 8 M, Na-phosphate 100 mM, Tris-HCl 10 mM, pH 8.0) for 50 min at room temperature and then centrifuged at 16,000 x g for 5 min at room temperature. Binding, refolding and elution using an AKTA Explorer system has been previously described [60].

### RT-QuIC assay

Real time QuIC was performed as described [60–62]. Briefly, reactions were set up in assay buffer containing 20 mM sodium phosphate (pH 6.9), 300 mM NaCl, 1 mM EDTA, 10 μM Thioflavin T and 0.1 mg/ml rPrP substrate. Ninety-eight μl aliquots were added to the wells of a 96 well optical bottom plate (Nalge Nunc International). Quadruplicate reactions were seeded with 2 μl of brain homogenate (10%) from CWD-negative animals or CWD-WTD and mCWD-WTD isolates that were 10-fold serially diluted in RT-QuIC seed dilution buffer (20 mM sodium phosphate (pH 6.9), 130 mM NaCl, 0.1% (w/v) SDS, 1X N2 Supplement (Invitrogen)). The plate was sealed with Nunc Amplification Tape (Nalge Nunc International) and placed in a BMG Labtech FLUOstar Omega fluorescence plate reader that was pre-heated to 42°C for a total of 50 hours with cycles of 1 minute double orbital shaking (700 rpm) incubation and 1 minute resting throughout the incubation. ThT fluorescence signals of each well were read and documented every 15 minutes then the values were plotted as the average of quadruplicate reactions by using GraphPad Prism software.

### Primary cell cultures

CGN were mechanically extracted from the cerebella of 5 to 7-day-old tg1536^+/+^ mice and enzymatically dissociated, as previously described [63]. Briefly, cells were plated at a density of 1.9 x 10^3^ cells/mm^2^ on plastic culture wells precoated with 10 μg/ml poly-D-lysine (Sigma-Aldrich, Ca). Cells were cultured in Dulbecco’s modified Eagle’s medium-Glutamax I high glucose (DMEM) (Life Technologies-Gibco, Ca) supplemented with penicillin and streptomycin (Life Technologies, Ca), 10% fetal bovine serum (Life Technologies, Ca), 20mM KCl (Sigma-Aldrich), and N2 and B27 supplements (Life Technologies, Ca). Cells were incubated at 37°C in a humidified 5% CO_2_ atmosphere. Every week, the medium was supplemented with glucose (1 mg/ml); in addition, the antimitotics uridine and fluorodeoxyuridine (10 μM) (Sigma-Aldrich) were added to reduce astrocyte proliferation. CWD-negative brain homogenate or WTD (3 independent experiments) and mWTD brain homogenates (one experiment) were added to CGN cultures as described [63, 64]. Briefly, brain homogenates were sonicated and added at a final concentration of 0.01% to CGN cultures 48 h after plating. Four days later, the medium was removed from the cultures, and cells were washed twice in fresh culture medium. Fresh medium was then added, and no medium changes were performed for the remaining experiments. On different days post infection (dpi), cells were washed twice with PBS and then incubated in lysis buffer (50 mM Tris-HCl [pH 7.4], 0.5% Triton X-100, 0.5% sodium deoxycholate) (Sigma-Aldrich, Ca) for 10 min at 4°C. The protein concentration of each cell lysate was measured with the bicinchoninic acid (BCA) protein assay (ThermoFisher Scientific-Pierce, Ca). Next, as previously described [63, 64], 50 μg of protein were digested with 5μg/ml of PK (VWR, Ca) for 30 min at 37°C, and the reaction was stopped by adding 1X pefabloc proteinase inhibitor to the mixture. Proteins were precipitated by the addition of methanol for 1 h at -20°C. The samples were then centrifuged at 16,000 xg (5417R rotor; Eppendorf) for 20 min and submitted to Western blot.

### Building a structural model for MD simulations

Using the SWISS-MODEL homology modelling server the initial model was generated [65]. The white-tailed deer PrP 112–233 sequence was uploaded and a template search was performed based on the SWISS-MODEL template library (SMTL), a template search was performed. A total of 201 templates were produced by the homology modelling search. Out of these, the crystal structure of deer prion protein (PDB: 4YXH [66]) model 1, chain A was chosen as the best template with a sequence identity of 99.01%, GMQE (Global Model Quality Estimation) score of 0.78 and a QMEAN4 score of -0.10. Finally, the SWISS-MODEL homology modelling server was used to build the 3D model [65].

### Preparing the model for simulation

Since the template structure and therefore the subsequent model lacked the N-terminal sequence “MKHV**G**GAAAAGAVVGG”, including the site of the 116G polymorphism (in bold), and two C-terminal residues “GA”, the Accelrys VS [67] software was used to add these amino acids to the model. Initial minimizations and equilibrations were run to relax the structure and allow the added peptide chain to fold naturally at 310 K for 10 ns. The complete construct 112–233 of wild type WTD prion generated by Accelrys VS [67] and equilibrated with MD was used as the control model. Finally, using the SWISS-MODEL homology modelling server, the model for the 116G polymorphism was generated based on the PDB file of the wt PrP structure (PDB: 4YXH [66]).

### Molecular dynamics simulations

The wt and 116G polymorphism systems were subjected to minimizations, equilibrations and production MD simulations with the Gromacs v 4.6.3 package [68] and the OPLS (Optimized Potential for Liquid Simulation) forcefield [69]. To minimize each system’s energy, the steepest decent method was used. Then a single point charge extended (SPC/E) rectangular periodic water box was used to solvate the models. In order to keep the system neutral Cl^−^ or Na^+^ ions were added. Next, energy minimizations with decreasing restraints on non-hydrogen protein atoms (Kposre = 1×10^5^, 1×10^4^, 1000, 100, 10 and 0 kJ mol^−1^ nm^−2^) were made to relax first the solvent, then the protein. Heating to 310K (Berendsen thermostat) and isotropic temperature coupling (NVT) equilibration to adjust solvent density to 1g/cm^3^ followed the minimizations. Lastly, equilibration steps and production runs were executed with NPT ensembles at 310K temperature and 1 atm pressure. The short-range electrostatic and van der Waals interactions cut-off radii were equal to 14 Å each. Long-range electrostatic interactions were treated with the particle-mesh Ewald method (PME), and all bond lengths were constrained with the LINCS algorithm with a fourth order of expansion. The production simulations were performed for 50 ns for each system. For both wt and 116G, two additional simulations using the same starting positions, but with different starting velocities of the atoms were run.

### Post-analysis tools

To analyze the MD trajectories, radius of gyration, root-mean-square deviations (RMSDs) and root-mean square fluctuations (RMSFs) were calculated for the Cα atoms using the g_gyrate, g_rmsd, and g_rmsf programs of GROMACS [68]. The solvent accessible surface areas and the hydrophobic surface areas were also calculated using the scripts implemented in GROMACS [68]. Assignments of the secondary structure content and the snapshots of trajectories or graphical representation of the models were done in VMD [70].

### PCR and DNA sequence analysis

Genomic DNA was extracted from brain homogenates using a commercial kit (DNeasy Blood and Tissue Kit (Qiagene) according to the manufacturer’s instructions. Primers were designed using software “Primer3Plus” (http://www.bioinformatics.nl/cgi-bin/primer3plus/primer3plus.cgi/) to amplify the sequence encoding mature cervid PrP (residues 24–234) with high specificity by PCR. The forward and reverse primers recognize the N-terminal (5’CCTAGTTCTCTTTGTGGCCATGTG3’) and C-terminal signal sequences (5’TGAGGAAAGAGATGAGGAGGATCAC3’) of PrP, respectively. The primers were synthesized at the University of Calgary CoreDNA service. The PrP sequence of WTD isolates from CWD-infected animals revealed that one animal harboured the wild type PrP genotype (A), wt, and the second one was heterozygous at amino acid 116 encoding both A (wt allele) and G, 116AG (S1 Fig). The sequencing results were confirmed by cloning of the PCR products.

### Serial dilution of PrP^res^

Ten percent brain homogenate prepared in PBS from WTD or mWTD isolates were treated with 50μg/ml of PK for one hour at 37°C and the reaction was terminated by adding 1X pefabloc proteinase inhibitor. The samples were then serially diluted and run analysed by Western blot using mAb 4H11 (S3 and S6 Figs).

## Supporting information

S1 Fig Sequencing results of WTD original isolates. WTD *Prnp* sequencing revealed a polymorphism at codon 116. At residue 116, the wt animal harbors GCA encoding alanine, whereas the second sequence encodes GCA and GGA indicative of heterozygosity for alanine and glycine at codon 116.
(TIF)

S2 Fig Conformational stability of WTD-wt. Conformational stability of an additional wt WTD isolate was assessed by densitometric analysis of PrP^res^ signals after guanidine denaturation.
(TIF)

S3 Fig PrP^res^ serial dilution of WTD isolates. Brain homogenate dilutions of wt (left panel) and 116AG (right panel) (undiluted or 1/2, 1/5, 1/10, 1/20, 1/50 and 1/100 diluted in sample buffer) after PK digestion were analysed by Western blot. PrP^res^ was detected using the monoclonal antibody 4H11.
(TIF)

S4 Fig First passage of WTD isolates in deer mice tg1536^+/+^. Transgenic mice tg1536^+/+^ overexpressing wt deer PrP were inoculated with 20 ul of wt or 116AG brain homogenates (1%). Incubation times of animals inoculated with 116AG prions are prolonged compared with wt prions. ***P <0.001 statistical analysis was evaluated using log-rank (Mantel-Cox) test.
(TIF)

S5 Fig Neuropathology of tg1536^+/+^ mice infected with WTD isolates. (**a-c** and **e**) PrP^Sc^ aggregates in the brains of tg1536^+/+^ mice inoculated with either of the two WTD isolates showed a punctate and diffuse distribution. (**c-f**) Spongiosis shown by higher magnification of the boxes in panels **a** and **b**. Brains of one mouse of each group were analysed. The coronal sections were stained with anti-PrP monoclonal antibody BAR224.
(TIF)

S6 Fig PrP^res^ serial dilution of mWTD brain homogenates. Brain homogenate dilutions (neat or 1/2, 1/5, 1/10, 1/20, 1/50 and 1/100 diluted in sample buffer) after PK digestion were analysed by Western blot. PrP^res^ was detected using the monoclonal antibody 4H11. mWTD-wt (left panel) and -116AG (right panel).
(TIF)

S7 Fig PrP^res^ accumlation in CGN^tg1536+/+^ primary neurons inoculated with mWTD brain homogenates. Accumulation of PrP^res^ in CGN^tg1536+/+^ cultures exposed to mWTD brain homogenate (first passage) was assessed by Western blot. Kinetics of PrP^res^ accumulation after exposure to brain homogenates from terminally ill tg1536^+/+^ mice infected with mWTD-wt or -116AG at a final concentration of 0.01% (wt/vol) were determined in duplicate. Fifty micrograms of protein from cell lysates were digested with PK, and PrP^res^ was detected with monoclonal antibody 4H11. PrP^res^ accumulation was observed from 14 dpi to 28 dpi for the mWTD-wt isolate (left panel), up to 28 dpi for the -116AG isolate (right panel).
(TIF)

S8 Fig Secondary passage of mWTD prions in tg1536^+/+^ mice. Transgenic tg1536^+/+^ mice overexpressing wt deer PrP were inoculated with mWTD-wt or -116AG brain homogenates. Statistical analysis was evaluated using log-rank (Mantel-Cox) test.
(TIF)

S9 Fig Tabulated secondary structure content from individual MD simulations. The dominant secondary structure elements were determined for each residue in wt and 116G PrP for each of three individual MD simulations. The averages from the three simulations were used to generate the curve in Fig 7F.
(TIF)
