# Supplementary figures and images for: Destabilizing polymorphism in cervid prion protein hydrophobic core determines prion conformation and conversion efficiency
Source: PLoS Pathog. 2017 Aug 11;13(8):e1006553. doi: 10.1371/journal.ppat.1006553 (PMC5568445; doi:10.1371/journal.ppat.1006553)

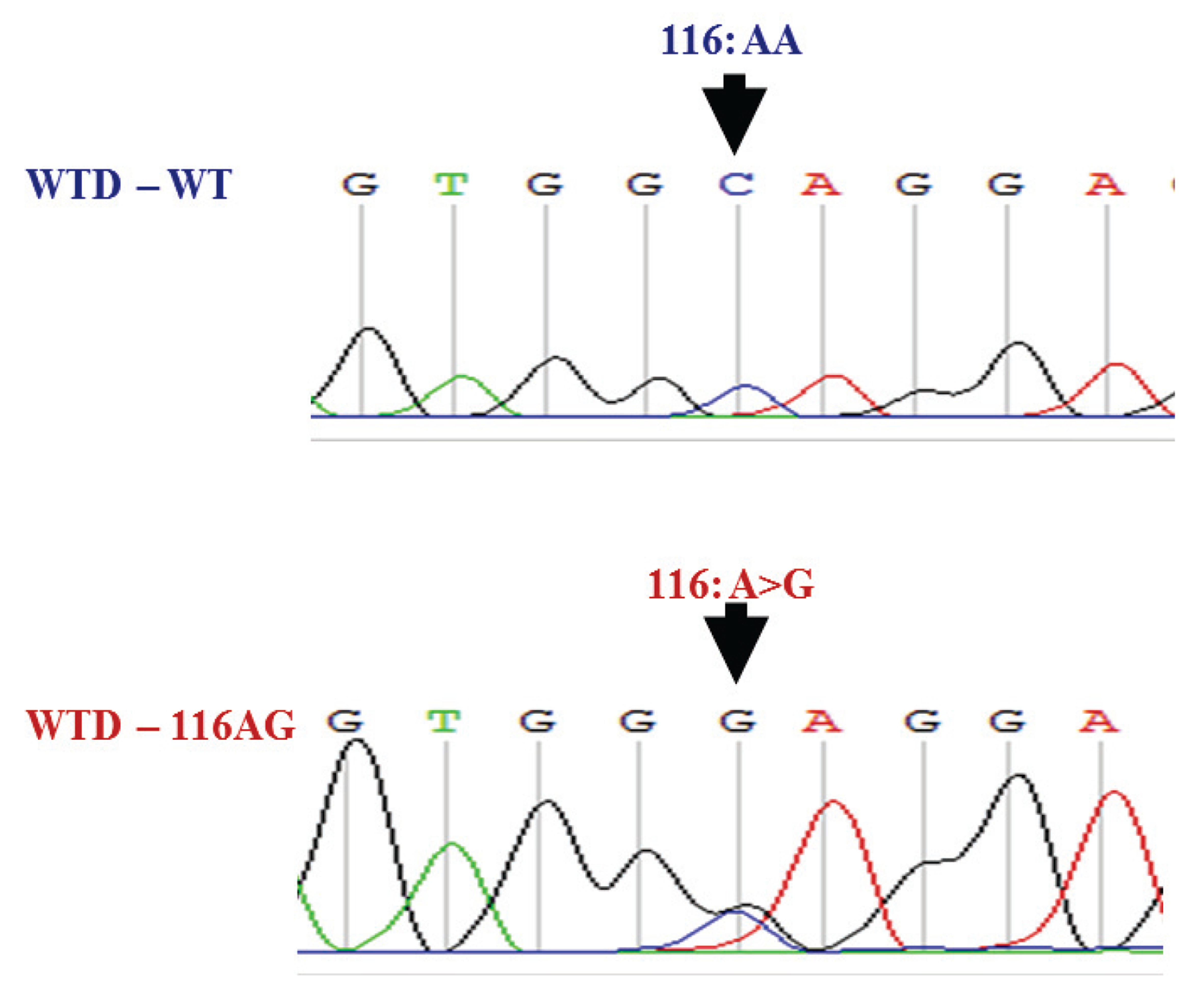

Supplement: S1 Fig — WTD Prnp sequencing revealed a polymorphism at codon 116. At residue 116, the wt animal harbors GCA encoding alanine, whereas the second sequence encodes GCA and GGA indicative of heterozygosity for alanine and glycine at codon 116. (TIF) [file ppat.1006553.s001.tif]

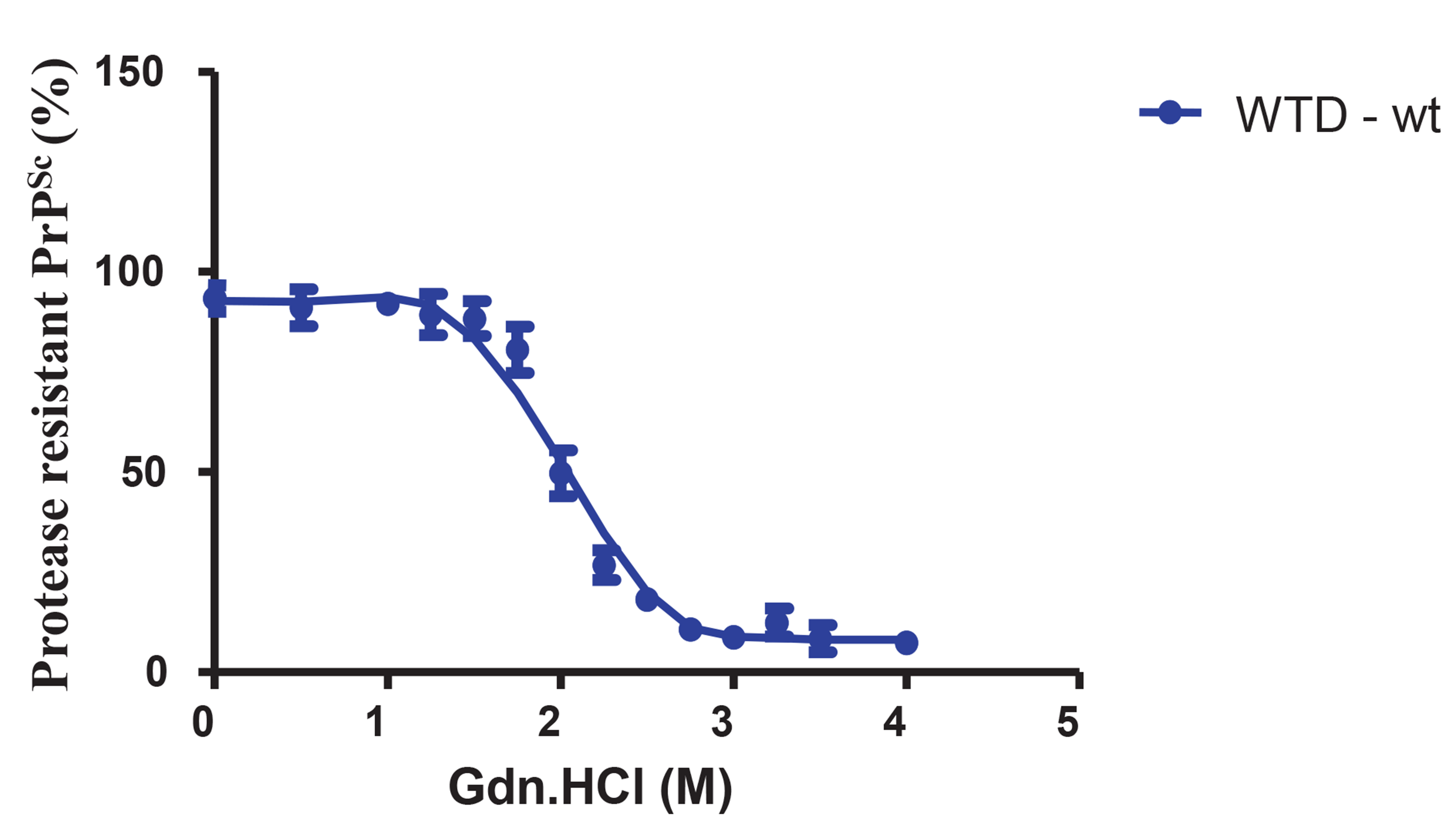

Supplement: S2 Fig — Conformational stability of an additional wt WTD isolate was assessed by densitometric analysis of PrPres signals after guanidine denaturation. (TIF) [file ppat.1006553.s002.tif]

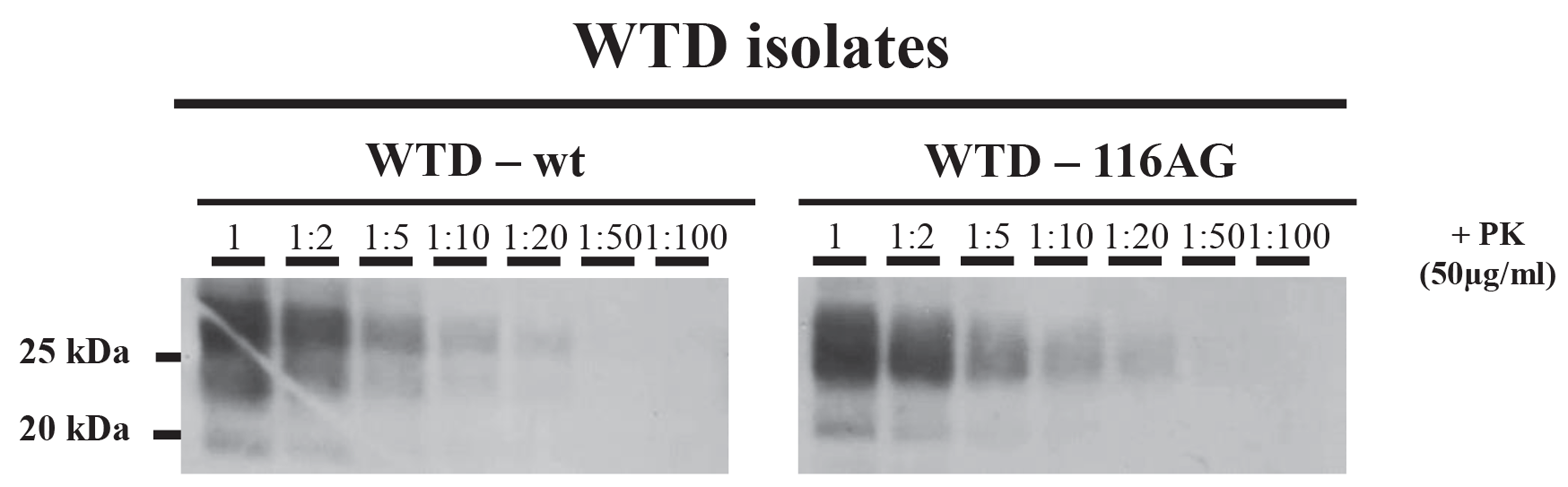

Supplement: S3 Fig — Brain homogenate dilutions of wt (left panel) and 116AG (right panel) (undiluted or 1/2, 1/5, 1/10, 1/20, 1/50 and 1/100 diluted in sample buffer) after PK digestion were analysed by Western blot. PrPres was detected using the monoclonal antibody 4H11. (TIF) [file ppat.1006553.s003.tif]

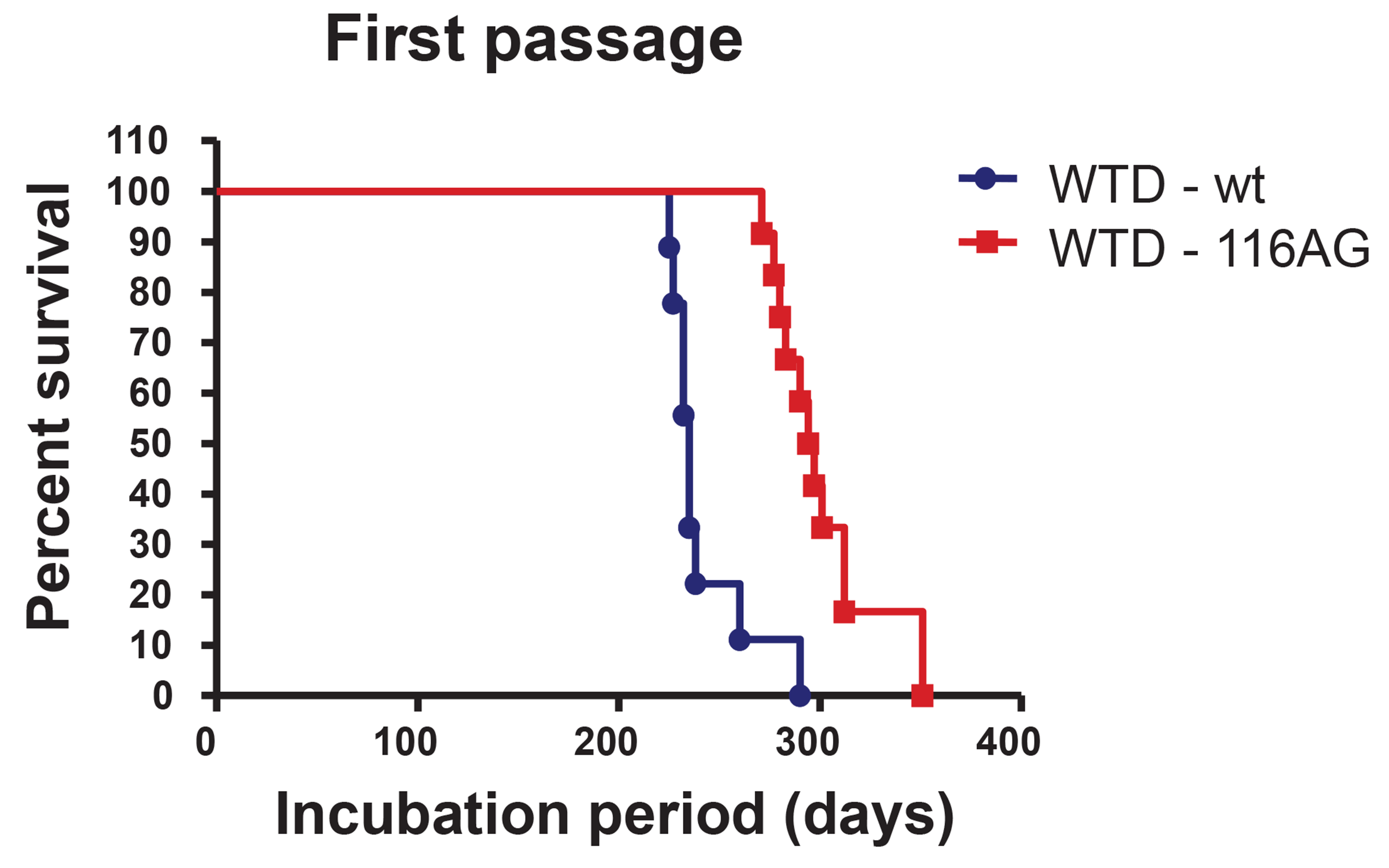

Supplement: S4 Fig — Transgenic mice tg1536+/+ overexpressing wt deer PrP were inoculated with 20 ul of wt or 116AG brain homogenates (1%). Incubation times of animals inoculated with 116AG prions are prolonged compared with wt prions. ***P <0.001 statistical analysis was evaluated using log-rank (Mantel-Cox) test. (TIF) [file ppat.1006553.s004.tif]

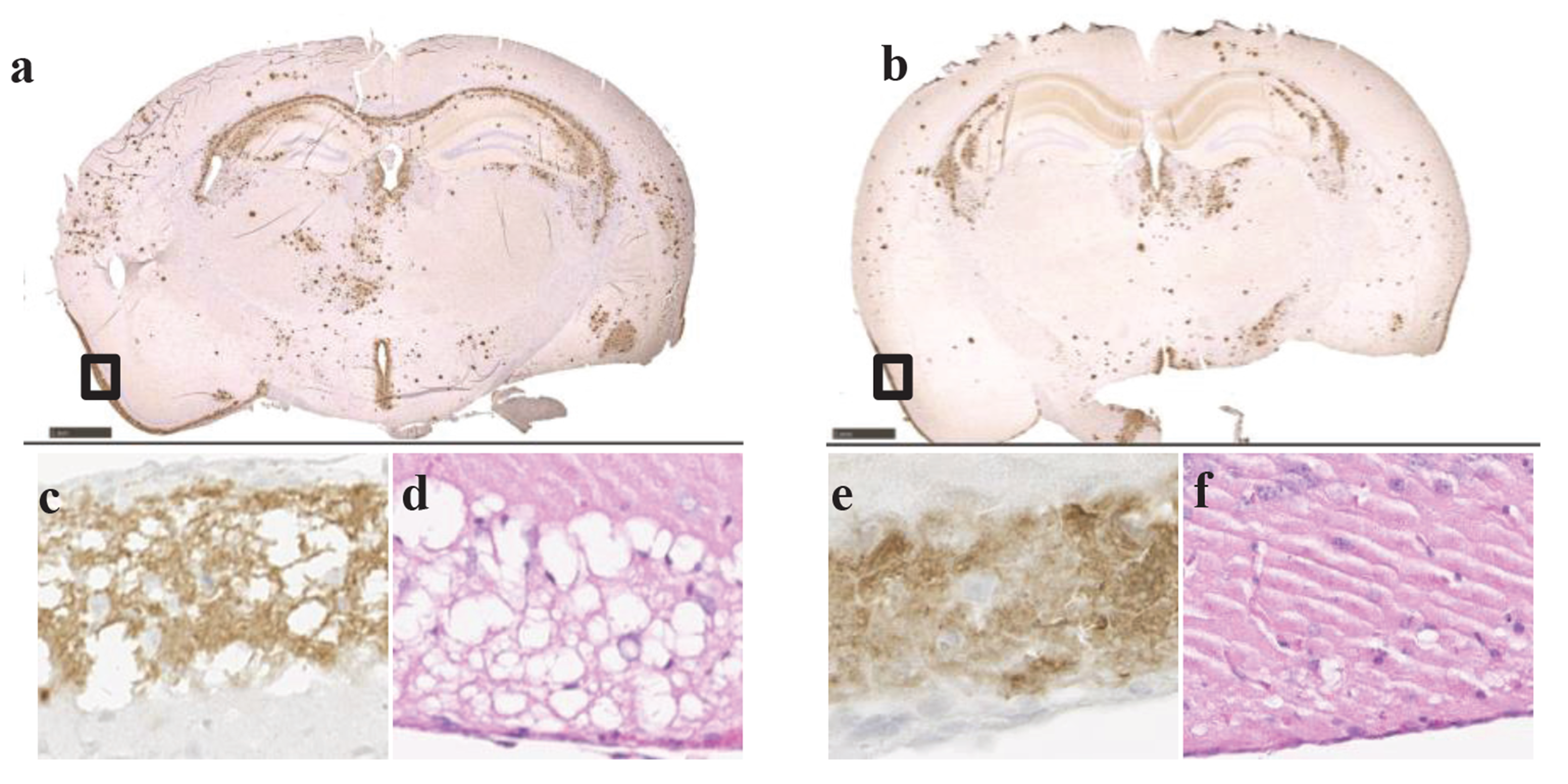

Supplement: S5 Fig — (a-c and e) PrPSc aggregates in the brains of tg1536+/+ mice inoculated with either of the two WTD isolates showed a punctate and diffuse distribution. (c-f) Spongiosis shown by higher magnification of the boxes in panels a and b. Brains of one mouse of each group were analysed. The coronal sections were stained with anti-PrP monoclonal antibody BAR224. (TIF) [file ppat.1006553.s005.tif]

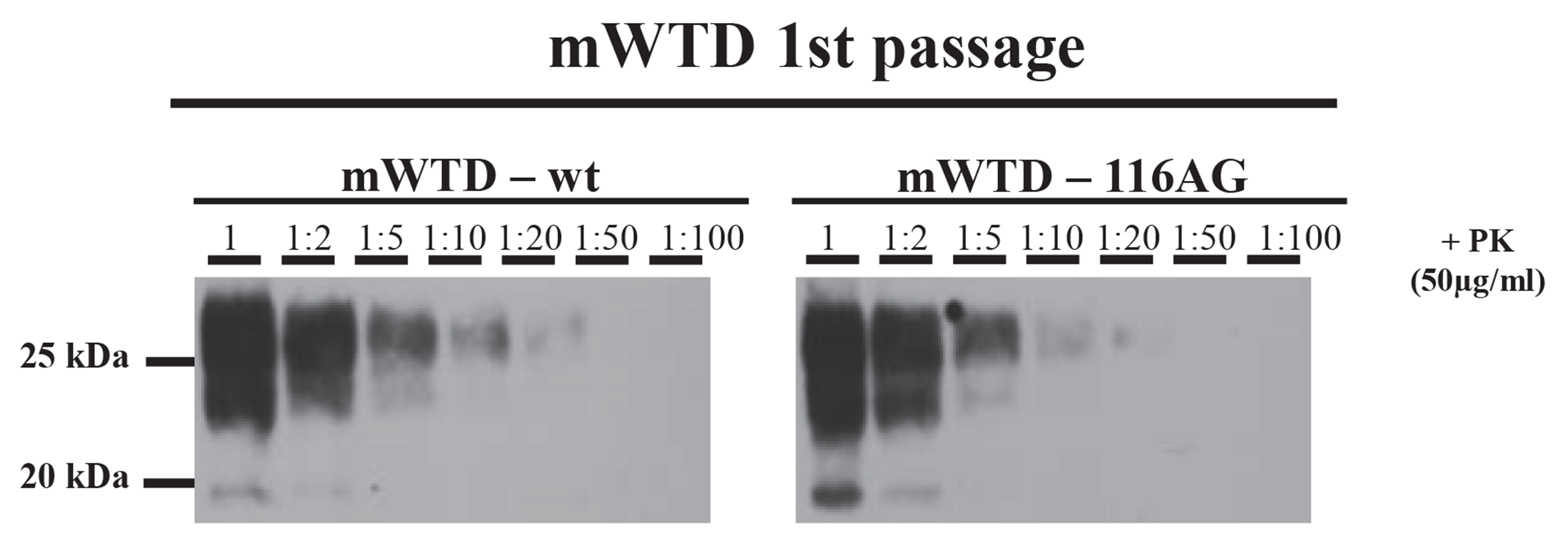

Supplement: S6 Fig — Brain homogenate dilutions (neat or 1/2, 1/5, 1/10, 1/20, 1/50 and 1/100 diluted in sample buffer) after PK digestion were analysed by Western blot. PrPres was detected using the monoclonal antibody 4H11. mWTD-wt (left panel) and -116AG (right panel). (TIF) [file ppat.1006553.s006.tif]

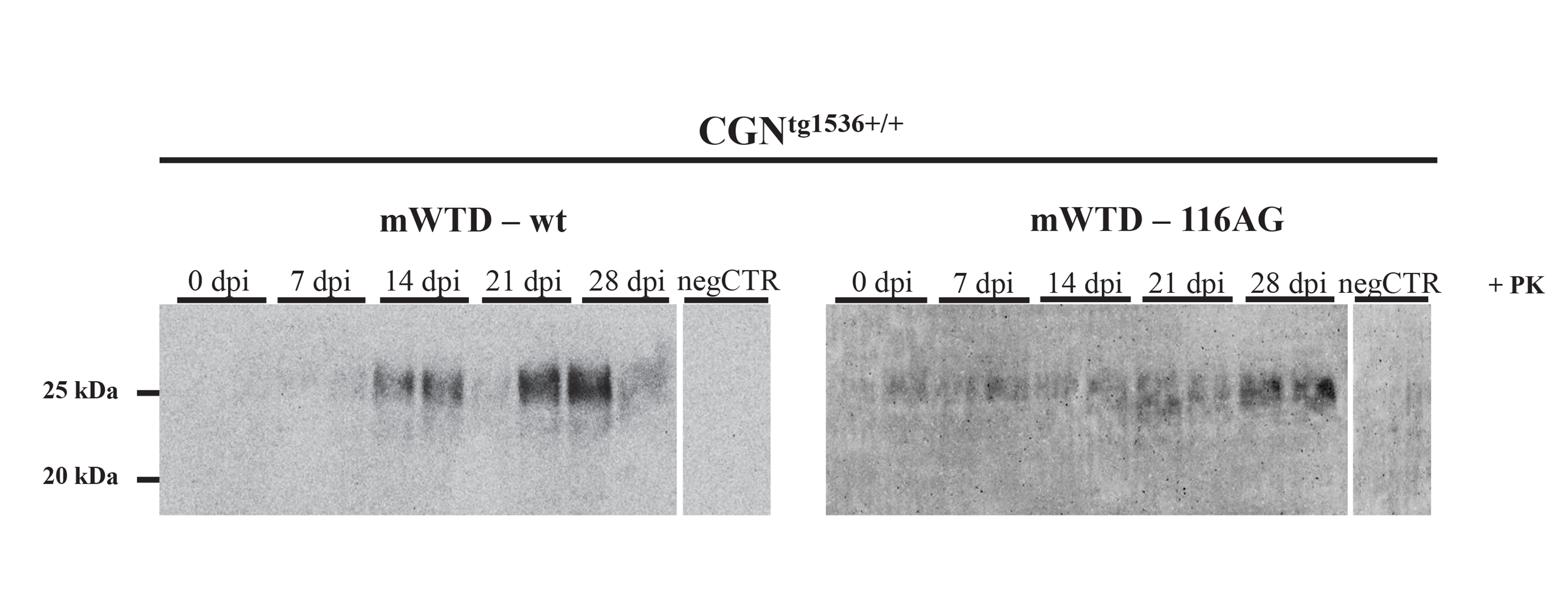

Supplement: S7 Fig — Accumulation of PrPres in CGNtg1536+/+ cultures exposed to mWTD brain homogenate (first passage) was assessed by Western blot. Kinetics of PrPres accumulation after exposure to brain homogenates from terminally ill tg1536+/+ mice infected with mWTD-wt or -116AG at a final concentration of 0.01% (wt/vol) were determined in duplicate. Fifty micrograms of protein from cell lysates were digested with PK, and PrPres was detected with monoclonal antibody 4H11. PrPres accumulation was observed from 14 dpi to 28 dpi for the mWTD-wt isolate (left panel), up to 28 dpi for the -116AG isolate (right panel). (TIF) [file ppat.1006553.s007.tif]

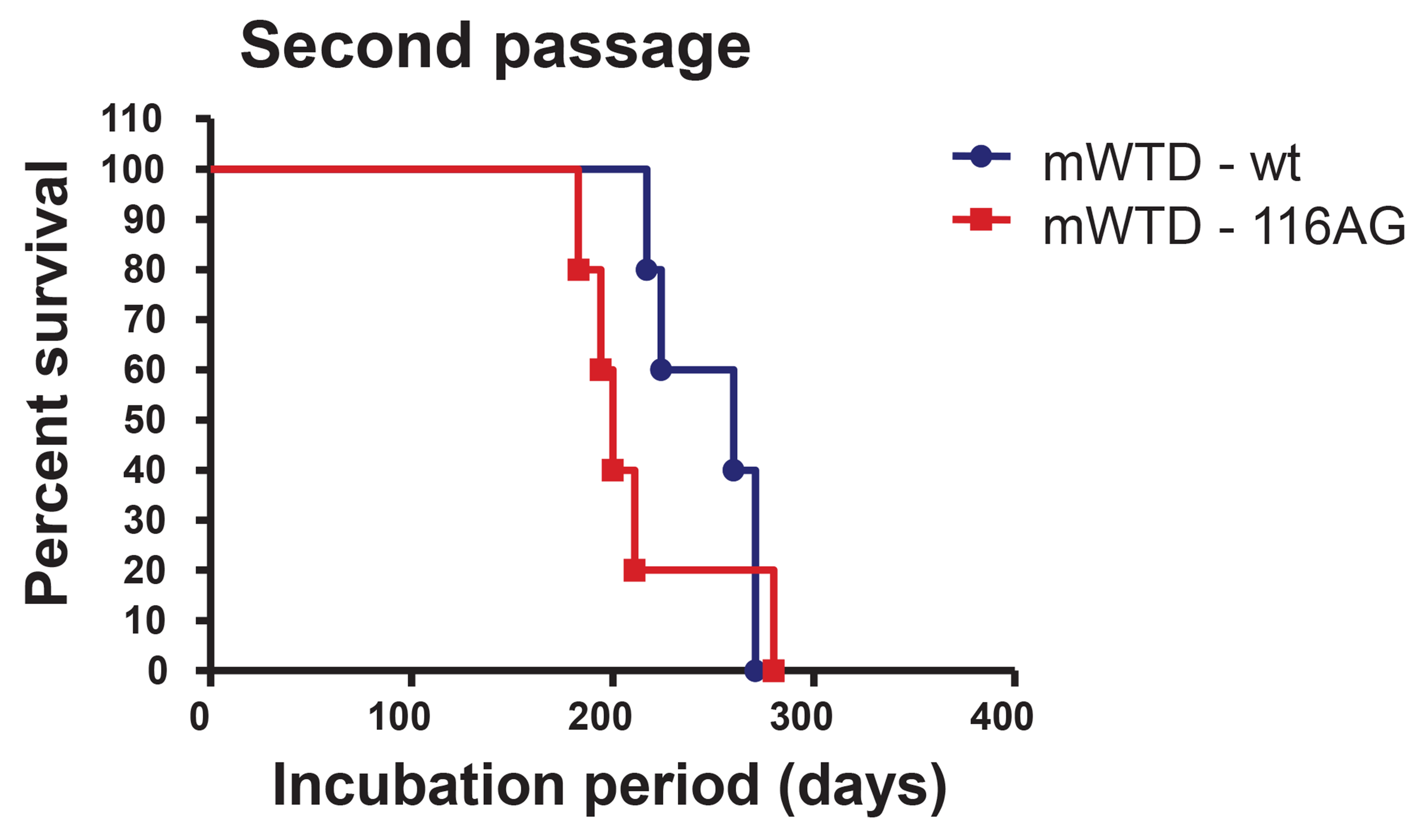

Supplement: S8 Fig — Transgenic tg1536+/+ mice overexpressing wt deer PrP were inoculated with mWTD-wt or -116AG brain homogenates. Statistical analysis was evaluated using log-rank (Mantel-Cox) test. (TIF) [file ppat.1006553.s008.tif]

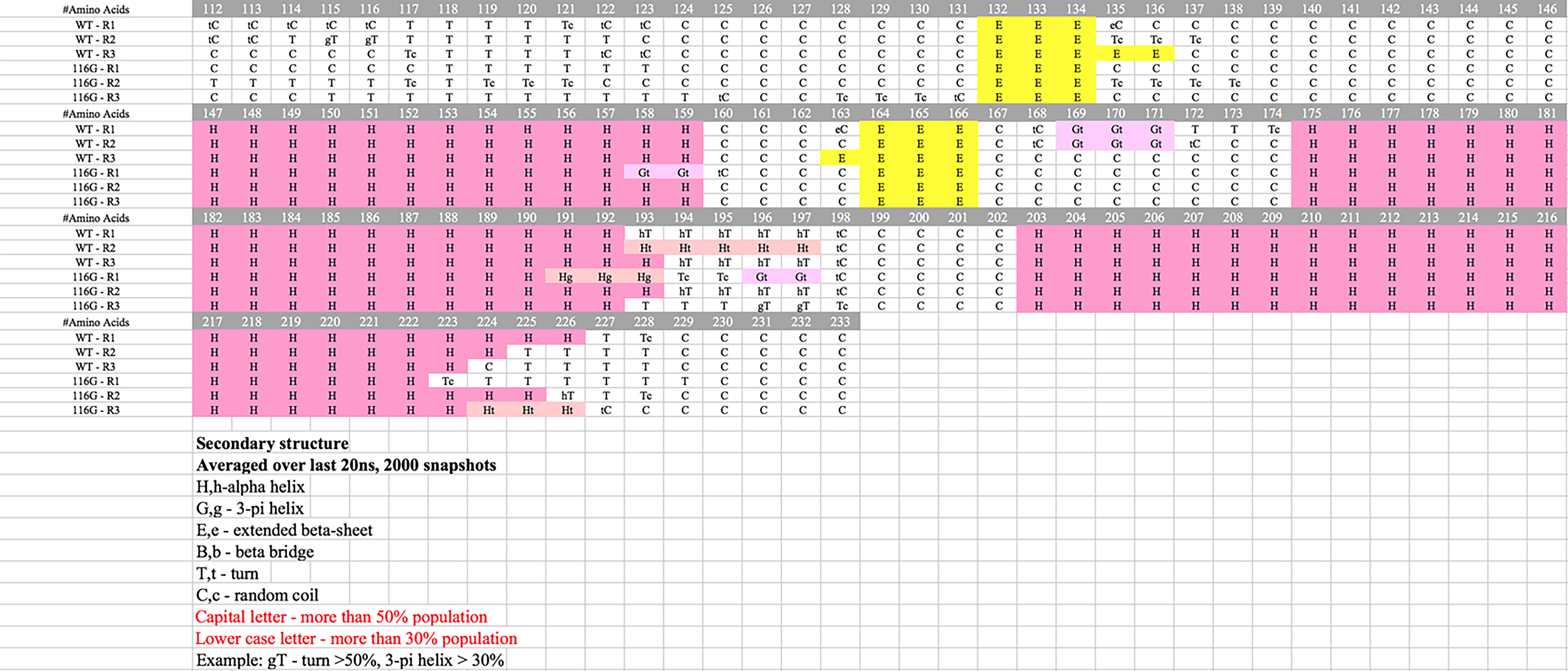

Supplement: S9 Fig — The dominant secondary structure elements were determined for each residue in wt and 116G PrP for each of three individual MD simulations. The averages from the three simulations were used to generate the curve in Fig 7F. (TIF) [file ppat.1006553.s009.tif]
